# A combination of linalool and linalyl acetate synergistically alleviates imiquimod-induced psoriasis-like skin inflammation in BALB/c mice

**DOI:** 10.3389/fphar.2022.913174

**Published:** 2022-08-05

**Authors:** Vineet Kumar Rai, Debabrata Chanda, Chandan Singh Chanotiya, Narayan Prasad Yadav

**Affiliations:** ^1^ Bio-prospection and Product Development Division, CSIR-Central Institute of Medicinal and Aromatic Plants, Lucknow, U. P., India; ^2^ Phytochemistry Division, CSIR-Central Institute of Medicinal and Aromatic Plants, Lucknow, U. P., India

**Keywords:** psoriasis, imiquimod, topical, PASI, synergistic activity, linalool, linalyl acetate, lavender oil

## Abstract

**Introduction:** Psoriasis is a chronic inflammatory skin disorder characterized by keratinocyte hyperproliferation and differentiation with increased immune cell infiltration. The anti-psoriatic effect of lavender oil has been reported. However, its phytoconstituents, linalool (L) and linalyl acetate (LA), showed a distinctive affinity with psoriasis targets.

**Objectives:** This investigation was aimed to determine the combined effect of L and LA in ameliorating psoriasis-like skin inflammation and its safety in long-term topical uses.

**Methods:** The combined effect of L and LA was compared with their individual effects. The anti-psoriatic activity was performed using imiquimod (IMQ)-induced psoriasis in BALB/c mice and evaluated to reduce PASI and CosCam scores and Th-1 and Th-17 cell-specific cytokine levels. The acute and repeated dose dermal toxicities were investigated as per the OECD guidelines.

**Results:** L and LA combination (LLA) in the 1:1 w/w ratio at 2% concentration showed a synergistic effect. The combination showed 76.31% and 71.29% recovery in PASI and CosCam Scores; however, L2% and LA2% showed 64.28% and 47.61% recovery in PASI and 64.75 and 56.76% recovery in CosCam scores, respectively. It showed >90% and >100% recovery in Th-17 and Th-1 cell-specific cytokines, respectively, and restored epidermal hyperplasia and parakeratosis toward normal compared with psoriatic mice. A marked reduction in NF-κB, cck6, and the IL-17 expression was also observed in the LLA-treated group. This combination was safe in a therapeutically effective dose for 28 days as no significant changes were observed in organ and body weights, liver and kidney parameters, and differential leukocyte counts.

**Conclusion:** This study proves the synergy between L and LA in a 1:1 w/w ratio at 2% in the treatment of psoriasis-like skin inflammation and provides strong scientific evidence for its safe topical use.

## 1 Introduction

Psoriasis is an immune-mediated disorder of the skin that lasts for several years. It has been characterized by hyper keratinocyte proliferation, leukocyte infiltration, and hyper cytokine expression (Di Meglio et al., 2014; Sharma et al., 2020). As it is an auto-immune disease, it is considered difficult to cure (Roy et al., 2019). However, getting rid of the psoriasis-associated symptoms is a first line strategy as far as the treatment of psoriasis is concerned (Who, 2016).

Lavender oil (LO) is an aromatic oil, i.e., extracted from *Lavandula angustifolia* Mill (Lamiaceae). It has immense utility as an aromatherapy massage oil, due to its anti-inflammatory and wound healing properties and providing relief in other skin conditions such as psoriasis, dermatitis, and eczema. Linalool and linalyl acetate are the major volatile components of the essential oils of several aromatic species including lavender. These compounds have been reported to possess various pharmacological properties. The anti-inflammatory effect of linalool is reported against ovalbumin-induced pulmonary inflammation (Kim et al., 2019), LPS-induced inflammation in BV2 microglia cells by activating Nrf2 (Li et al., 2015), cigarette smoke-induced lung inflammation by inhibiting NF-κB activation (Ma et al., 2015), and lipopolysaccharide-induced lung inflammation (Huo et al., 2012). Recently, linalyl acetate has been reported to recover the cell damage and cardiovascular changes caused by an acute nicotine-induced cardiovascular disruption in adolescent rats (Kim et al., 2017). However, a report published in 2002 by Peana et al. (2002) exhibits the anti-inflammatory effect of linalool and linalyl acetate against carrageenan-induced paw edema in mice. Additionally, linalool has shown a good psychopharmacological effect in mice, revealing its marked dose-dependent sedative effects on the central nervous system as well (Buchbauer et al., 1991; Jirovetz et al., 1991), including protection against pentylenetetrazol, picrotoxin and transcorneal electroshock-induced convulsions, hypnotic and hypothermic properties (Elisabetsky et al., 1995; Elisabetsky et al., 1999). Another study exhibited that it modulates the expression of glutamate activation *in vitro* (competitive antagonism of L-[^3^H] glutamate binding) and *in vivo* (delayed subcutaneous N-methyl D-aspartate-induced convulsions and blockade of intracerebroventricular quinolinic acid-induced convulsions) (Brum et al., 2001; Silva Brum et al., 2001). Anesthetic activity related to its effects on the nicotinic receptor-ion channel (Ghelardini et al., 2000), spasmolytic effect (Lis-Balchin and Hart, 1999), and antimicrobial activity against several bacteria and fungi (Carson and Riley, 1995; Pattnaik et al., 1997) are the most vital outcomes related to linalool’s pharmacological effects. Moreover, linalool, as well as other terpenes and terpenoids, could enhance the permeability of a number of drugs through skin or mucus membranes (Kunta et al., 1997; Ceschel et al., 1998; Kommuru et al., 1998). For the first time, we reported the anti-psoriatic potential of lavender oil (LO) and its major phytoconstituents, i.e., linalool (L) and linalyl acetate (LA) against imiquimod-induced psoriasis. It was interesting to observe that the linalool and linalyl acetate exhibited anti-psoriatic action by different mode of actions. Linalool showed more than 50% recovery in PASI scores as well as in the levels of Th-17 cell cytokines (IL-17 and IL-22); however, linalyl acetate showed good recovery (more than 90%) in the levels of Th-1 cytokines (TNF-α and IL-1β), specifically at 2% topical dose (Rai et al., 2020). These findings suggested us to investigate the combined effect of linalool and linalyl acetate. Therefore, in the present investigation, different combinations of L and LA were compared for their anti-psoriatic effect in different ratios. During the chemical analysis (GC and GC-MS) of lavender oil, we observed that linalool and linalyl acetate are present in 3:4 ratio approximately (14.2% and 20.0% respectively). Therefore, the anti-psoriatic activity of linalool and linalyl acetate combination was tested in the ratio of 1:4, 2:3, 1:1, 3:2, and 4:1.

As psoriasis expresses both (Th-17 and Th-1) types of cytokines, the combined effect of L and LA, whether additive or synergistic, could be better utilized for the comprehensive treatment of psoriasis. Therefore, we investigated the combined effect of L and LA on the comprehensive treatment of psoriasis and their toxicity studies to ensure its clinical pertinence for human use and to provide a safe and effective alternative to the current therapy.

## 2 Materials and methods

### 2.1 Materials

Materials used in this study were purchased in India. Cyclophosphamide, linalool, linalyl acetate, and albumin 5% solution were from Sigma Aldrich; Imiquimod (IMQ) cream (5%) was the product of Glenmark Pharmaceuticals Ltd.; Polyethylene glycol 200 (PEG200), alcohol, hematoxylin, eosin, DPX mountant, and xylene were from Thomas Baker; Paraffin wax (60–62°C) was from Merck; ELISA Kits (TNF-α, IL-1β, and IL-6) were from Invitrogen Bio-services; IL-17 A and IL-22 were from Koma Biotech; NF-κB, IL-17, and cck6 specific primary and secondary antibodies were from Santa Cruz, and hydrogen peroxide (H_2_O_2_; 30%) and DAB chromogen were from TCI Chemicals. The solvents and chemicals used in the study were of analytical grade. We used Milli-Q water throughout the study.

The product specification and description of the linalool (Catalog number W263516) and linalyl acetate (Catalog number W263613) are given in Supplementary Material S1, Supplementary Figure S1; Buchbauer et al. (1991). The combination of the phyto-molecules used in this investigation is endotoxin-free.

### 2.2 Ethics statement

Either sex of BALB/c mice (25–30 g) and Wistar rats (220–240 g) were obtained from the animal house facility “Jeevanika” of the institute. Animals were housed and acclimatized under controlled laboratory conditions (25°C ± 3°C room temperature and 60% humidity along with 12 h light and 12 h dark cycle) with free access to food and water *ad libitum* before starting the actual experiment*.* Experimental protocols for anti-inflammatory activity (AH-2012-05), acute dermal toxicity study (AH-2012-01), and repeated dose dermal toxicity study (AH-2012-01) were duly approved by the Institutional Animal Ethics Committee (400/01/AB/CPCSEA), Government of India. Animal experiments were conducted following the principles for laboratory animal use and care found in European Community guidelines (EEC Directive of 1986; 86/609/EEC). The safety assessments were commenced in a single trial based on the Organization for Economic Cooperation and Development (OECD) guidelines 404 and 410.

### 2.3 Methods

#### 2.3.1 *In vivo* experiments

##### 2.3.1.1 Assessment of the anti-psoriatic activity of LLA

Psoriasis-like skin lesions in BALB/c female mice were induced by topical administration of IMQ. Induction of the disease and dosing was carried out as per the procedure described in our previous study (Rai et al., 2020). Test control was taken as a 2% w/v solution of linalool and linalyl acetate combination in different ratios (4:1, 3:2, 1:1, 2:3, and 1:4 w/w) in PEG200. About 100 µL of the test samples were applied daily to the mice’s skin. Psoriasis area and severity index (PASI) scores and CosCam scores were measured on the 0th, 2nd, 4th, 6th, and 8th days of the study period (Liu et al., 2019). Dorsal skin tissues were harvested for biochemical, immunohistochemical, and histological investigations on the final day.

##### 2.3.1.2 Cytokine’s estimation

The collected skin tissues were homogenized in phosphate buffer (10% homogenate). Homogenates were centrifuged (Sigma Laborzentrifugen 3K30) at 10,000 RPM/4°C for 15 min. Supernatants were taken and subjected to antigen-antibody reactions. Quantification of total protein (pg/mL), i.e., pro-inflammatory cytokines (IL-1β and TNF-α) and Th-17 cell cytokines (IL-17 A and IL-22), was performed as per the user’s manual of mouse Enzyme-Linked Immune Sorbent Assay (ELISA) kits (Mishra et al., 2016b).

##### 2.3.1.3 Immunohistochemistry

Immunohistochemistry (IHC) was performed to observe the expression of NF-κB, IL-17, and cck6 in the skin tissue sections as per the procedure reported elsewhere (Somagoni et al., 2015). Briefly, collected skin tissues were fixed in formalin and embedded in paraffin wax. About 5 µm thick sections were cut using a microtome; slides were prepared and hydrated using xylene, followed by different strengths of alcohol. Furthermore, tissues were treated with 3% H_2_O_2_ and 5% albumin and incubated at 4°C for 24 h with the primary antibodies against the corresponding markers. Horseradish peroxidase-conjugated secondary antibody was used to find the expression of the primary antibody. Tissues were treated with DAB chromogen and counterstained using hematoxylin. This process was carried out to produce brown staining, indicating activated markers’ presence. Finally, the specimens were observed under Olympus BX40 light microscope, i.e., equipped with a computer-controlled digital camera (DP71, Olympus Center Valley, PA). The intensity of the brown color produced was checked by the ImageJ.exe app. The statistical differences were calculated based on the relative intensity scores of each image.

##### 2.3.1.4 Histopathology

Skin tissue samples for histopathological analysis were kept in 10% formalin buffer at room temperature. Formalin buffered skin tissues were embedded in paraffin wax, cut into 7 µm sections using a microtome, stained with hematoxylin and eosin (H and E) dye, and observed at 10X magnification for histological changes using Leica DM 750 microscope. Histopathological changes, i.e., epidermal and dermal hyperplasia, para-keratosis, hyper-keratosis, dermal edema, vesicular formation, and granulosis, were evaluated and compared between the groups (Shah et al., 2012; Raza et al., 2013).

##### 2.3.1.5 Assessment of the anti-psoriatic effect

The anti-psoriatic effect of LLA was investigated by percentage reduction in the parameters (Yadav et al., 2008) like PASI scores, CosCam scores, and the levels of Th-1 and Th-17 cell expressing cytokines compared to DC group using Eq. (1).

$$\text{Anti}-\text{psoriatic}\;\text{ activity }\left(\text{\%}\right)=\;\frac{\mathrm{Value}\;\mathrm{of}\;\mathrm{toxin}\;\mathrm{group}-\mathrm{treated}\;\mathrm{group}}{\mathrm{Value}\;\mathrm{of}\;\mathrm{toxin}\;\mathrm{group}-\mathrm{control}\;\mathrm{group}\;}\times 100.\;$$ (1)

*Sample that showed >50% reduction in the parameters selected previously were considered active (Carlin et al., 2004).

##### 2.3.1.6 Acute dermal toxicity study

This investigation was carried out using OECD Test guideline no. 404 (adopted on 9 October 2017) (Oecd, 2015) as per the procedure reported by Wang et al. (2017) with modification. Healthy young adult BALB/c mice (25–30 g; either sex) and Wistar Rats (220–240 g; either sex) were used. Non-pregnant females were used in these experiments. Issued animals were acclimatized to the experimental environment for 7 days before the commencement of the investigation. The temperature and humidity in the experimental animal room were maintained at 22°C ± 3°C and 55% ± 5% RH, respectively. The artificial lighting of 12 h light and 12 h dark was maintained. Animals were provided *ad libitum* access to a commercial diet and drinking water. Animals (*n* = 6) were randomly selected and marked to provide individual identification. One day before the administration of the test sample, fur was removed from the dorsal/flank area of the animals (i.e., at least 10% of the total body surface area) by closely clipping with a curved scissor. Anesthesia was used to aid in handling animals and minimize animal stress if and when required. Utmost care was taken to avoid abrasion of the skin to avoid the alteration of the skin permeability. The clipped skin area was divided into two test sites of one square inch each. Normal saline was chosen as vehicle control. Test samples used for the investigation were LLA (1:1 w/w) at 2%, 10%, and 20% solutions in PEG 200. About 50 µL of these samples were applied once on one test site of the animal against the vehicle (50 µL). The sites were macroscopically examined on the 1st, 7th, and 14th days for skin irritation in terms of erythema and edema. Animals were observed for any casualty also. Skin reactions were graded separately, each time on a 0–4 grading scale, and the test materials were categorized based on primary irritation index values as reported elsewhere (Yadav et al., 2014; Mishra et al., 2016a). High-definition pictures of the animals were taken on the 1st, 7th, and 14th day for macroscopic examination of the skin surfaces. The body weight of the animals was taken on the last day of the study period. Organ weight (the heart, lung, liver, kidney, and spleen) was measured only in the case of mice. If and when a decrease in organ weight was observed in mice, rats could have also been sacrificed for organ weight measurements.

##### 2.3.1.7 Repeated dose dermal toxicity study

A repeated dose dermal toxicity study was carried out as per the OECD guideline 410 for testing of chemicals (adopted on 12 May 1981) (Oecd, 2018) as per the reported procedure by Djerrou et al. (2013) with modification. Healthy young adult Wistar rats (220–240 g; either sex) were employed in this study. Non-pregnant females were used in these experiments. Issued animals were acclimatized to the experimental environment for 7 days before the commencement of the investigation. Animals (*n* = 6) were randomly selected and marked to provide individual identification. On day before the administration of the test sample, the fur of the animals was removed from the dorsal/flank area (i.e., at least 10% of the total body surface area) by clipping with a curved scissor. Clipping was repeated, if needed, every week. Utmost care was taken to avoid the abrasion of the skin that led to the altered skin permeability. The clipped skin area was divided into two test sites of one square inch each. Normal saline was chosen as vehicle control. Test samples used for the investigation were LLA (1:1 w/w) at 0.2%, 2%, and 4% solutions in PEG 200. About 50 µL of the test samples and vehicle were applied once daily on one of the test sites of the animals against the vehicle for 28 days. The pictures of the animals were taken on the 1st, 7th, 14th, 21st, and 28th days for macroscopic examination of the skin surfaces. The body weight of the animals was taken on the 1st, 7th, 14th, 21st, and 28th days of the study period to monitor any significant variation in the weight. The organ (Heart, Lung, Liver, Kidney, and Spleen) weight of the animals was measured on the last day of the investigation, and the blood sample was collected from the retro-orbital route for different biochemical and hematological evaluations (Obara et al., 1997).

#### 2.3.2 Assessment of the intactness of linalool and linalyl acetate combination

##### 2.3.2.1 Gas chromatographic analysis

Varian CP-3800 Gas Chromatography analyzed linalool and linalyl acetate in a 1:1 w/w ratio. DB-5 capillary column of 30 m length, 0.25 mm internal diameter, and 0.25 µm film thickness was used for the analysis. The column oven temperature was programed with a rate of 3°C/min from 60°C to 240°C for 2 min hold time at 240°C. Hydrogen was used as a carrier gas with a constant flow rate of 1 mL/min in the split ratio of 1:40, and injector and detector (Flame Ionization Detector) temperatures were maintained at 280°C.

##### 2.3.2.2 NMR analysis

The intactness of linalool and linalyl acetate in combination was also confirmed by ^13^C and ^1^H 1D NMR by taking the spectra of linalool and linalyl acetate in a 1:1 w/w ratio. This investigation was performed to assess the possible interaction between linalool and linalyl acetate (López-Martínez et al., 2015). Spectra were produced using Bruker Advance spectrometer (Billerica, United States ) in CDCl_3_ at 301 K.

#### 2.3.3 Statistical analysis

Data are shown as means ± SEM. Data for treatment groups were compared using one-way analysis of variance (ANOVA) followed by Tukey’s post hoc test using GraphPad Prism (R), Version 5.01 (GraphPad software. Inc. United States). #*p* < 0.05, ##*p* < 0.01, and ###*p* < 0.001 (NC vs. DC), *p < 0.05, **p < 0.01, and ***p < 0.001 (DC vs. treatments).

## 3 Results

### 3.1* In vivo* anti-psoriatic efficacy of L and LA combination

**FIGURE 1:**
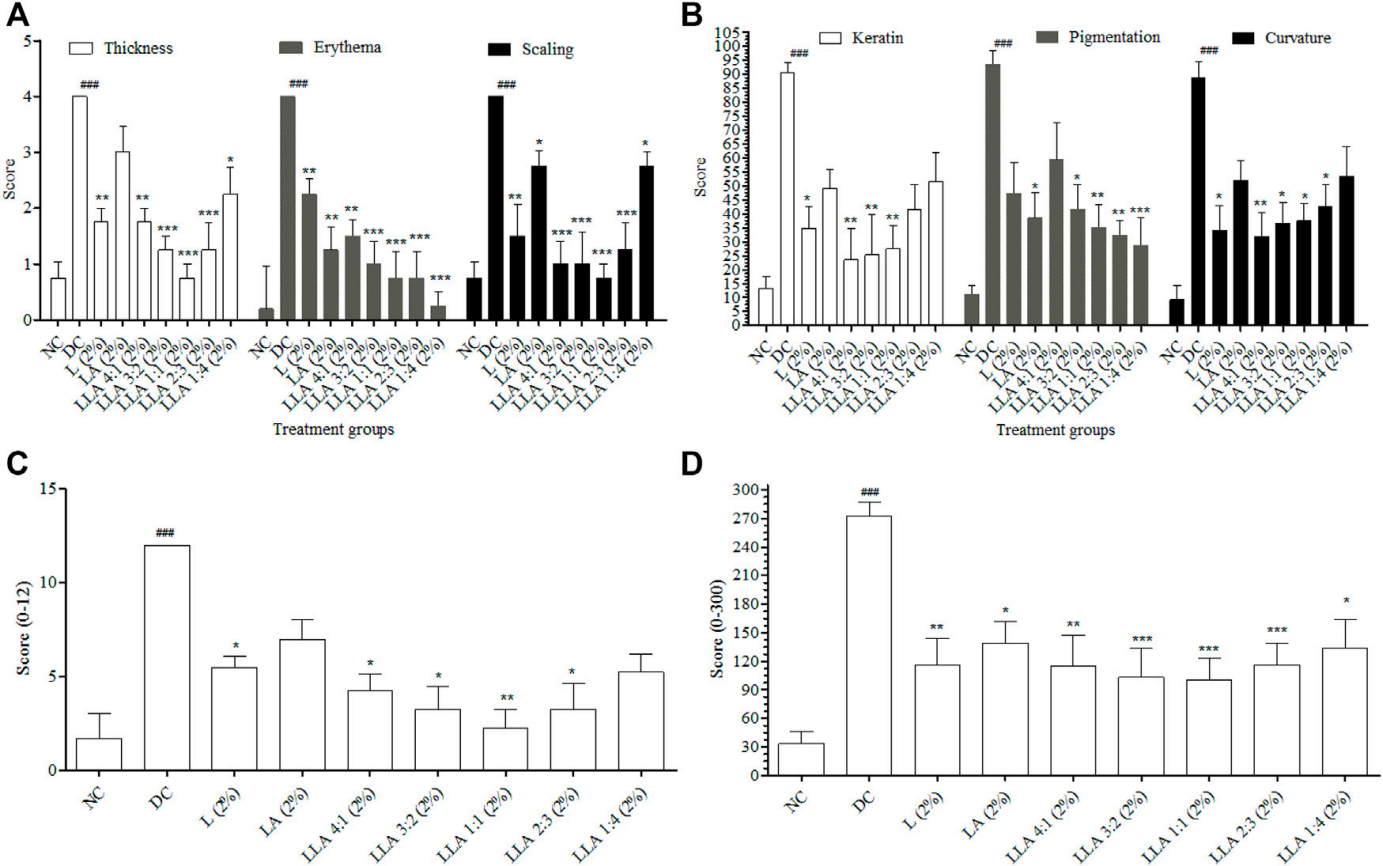
Results of PASI and CosCam scores of the mice treated with different ratios of LLA combination. Graph **(A)** depicts thickness, erythema, and scaling of PASI, **(B)** depicts keratin, pigmentation, and curvature of CosCam scores, **(C)** depicts cumulative PASI scores, and **(D)** depicts cumulative CosCam scores of the treatment groups. NC; normal control, DC; disease control, LLA (2%); linalool + linalyl acetate in 4:1, 3:2, 1:1, 2:3, and 1:4 ratios at 2% concentration. The data represent mean ± SEM, *n* = 6. ^###^ *p* < 0.001 (NC vs. DC), *p < 0.05, **p < 0.01, and ***p < 0.001 (DC vs. treatments).

PASI and CosCam parameters were scored to portray the severity of psoriasis-like inflammation. The visual manifestations like erythema, scaling, and thickness increased significantly in the DC group on the 8th day of the study (Data from the 2nd, 4th, and 6th days was not provided as no significant change in the level of erythema, scaling, and thickness was observed on these days). LLA showed significant alleviation in all the PASI and CosCam Parameters (Figure 1). LLA 2% (3:2 w/w) and LLA 2% (1:1 w/w) attained PASI 70 and PASI 75, respectively (Table 1).

**TABLE 1:** Anti-psoriatic effect of linalool (L) and linalyl acetate (LA) combinations.

	Recovery (%)							
Parameter		Linalool (2%)	Linalyl acetate (2%)	LLA 2% (4:1 w/w)	LLA 2% (3:2 w/w)	LLA 2% (1:1 w/w)*	LLA 2% (2:3 w/w)	LLA 2% (1:4 w/w)
PASI	Thickness	64.28 ± 6.18	21.42 ± 1.12	53.95 ± 3.54	67.12 ± 7.57	**80.26** ± 6.13	67.12 ± 6.88	40.78 ± 2.09
	Erythema	42.85 ± 3.27	85.71 ± 8.86	60.53 ± 3.25	73.68 ± 8.26	**73.68** ± 6.24	80.26 ± 9.20	86.84 ± 4.82
	Scaling	85.71 ± 7.40	35.71 ± 4.47	75.00 ± 6.49	75.00 ± 7.98	**75.00** ± 8.29	62.50 ± 4.31	50.00 ± 6.93
	PASI	**64.28** ± 6.75	**47.61** ± 4.81	63.16 ± 4.43	71.93 ± 7.94	**76.31** ± 6.89	69.96 ± 6.80	59.20 ± 4.61

CosCam	Keratin	75.76 ± 8.47	55.52 ± 7.28	84.96 ± 9.93	82.82 ± 9.06	**80.06** ± 5.37	62.88 ± 5.58	50.30 ± 2.94
	Pigmentation	52.14 ± 3.08	72.39 ± 5.58	39.57 ± 4.71	61.34 ± 7.70	**69.33** ± 8.40	72.70 ± 7.89	76.99 ± 6.45
	Curvature	66.35 ± 5.09	42.28 ± 3.79	71.29 ± 3.36	65.74 ± 7.81	**64.50** ± 6.03	58.02 ± 3.76	44.75 ± 3.20
	CosCam	**64.75** ± 5.55	**56.76** ± 5.56	65.27 ± 6.00	69.96 ± 8.19	**71.29** ± 6.60	64.53 ± 5.74	57.34 ± 4.19

Th-1 cell cytokines	TNF-α	70.86 ± 8.14	99.05 ± 11.05	79.97 ± 6.71	91.34 ± 8.40	**103.11** ± 7.03	104.42 ± 8.10	104.60 ± 7.77
	IL-1β	79.98 ± 9.30	94.33 ± 6.28	83.18 ± 4.16	92.48 ± 9.00	**97.65** ± 9.48	99.43 ± 5.00	99.53 ± 8.36

Th-17 cell cytokines	IL-17	73.39 ± 8.14	45.43 ± 6.54	87.65 ± 6.08	77.83 ± 6.28	**76.94** ± 5.40	58.09 ± 8.82	32.37 ± 5.04
	IL-22	83.07 ± 5.85	65.49 ± 6.99	98.56 ± 8.06	91.65 ± 9.36	**90.19** ± 6.40	86.42 ± 6.27	75.96 ± 5.55

LLA, linalool + linalyl acetate combinations; *consistent synergistic effect. The data represent mean ± SD, *n* = 6.

**FIGURE 2:**
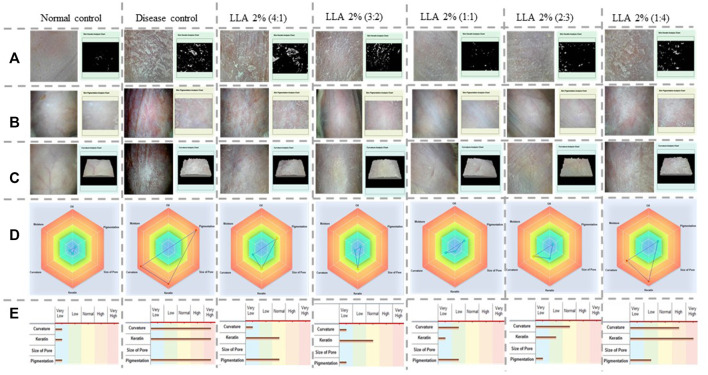
Pictorial representation of keratin, pigmentation, curvature analysis charts, radar, and bar graphs showing the severity of skin inflammation in different tested groups, Vertical columns present the different treatment groups, and horizontal rows denote **(A)** the keratin analysis chart, **(B)** the pigmentation analysis chart, **(C)** the curvature analysis chart, **(D)** the radar graph, and **(E)** bar graphs of the psoriatic and animals treated with linalool and linalyl acetate.

Figure 2 depicts different treatment groups with the skin condition of each group of mice, keratin analysis chart, pigmentation analysis chart, curvature analysis chart, radar graph, and bar graphs. As depicted by their respective analysis charts, a marked increase in keratin, pigmentation, and curvature was observed in psoriatic mice. Test control groups showed alleviation in these parameters. The highest reduction can be observed in the case of LLA 2% in 3:2, 1:1, and 2:3 ratios. As the size of the triangle of the radar graph directly reflects the severity of the skin inflammation, a smaller size reflects less intense inflammation. The smallest size of the triangle and bar was observed in the case of LLA 2% in 3:2, and 1:1 ratios. This shows the comprehensive utility of these combinations against psoriasis.

#### 3.1.1 Levels of cytokines

**FIGURE 3:**
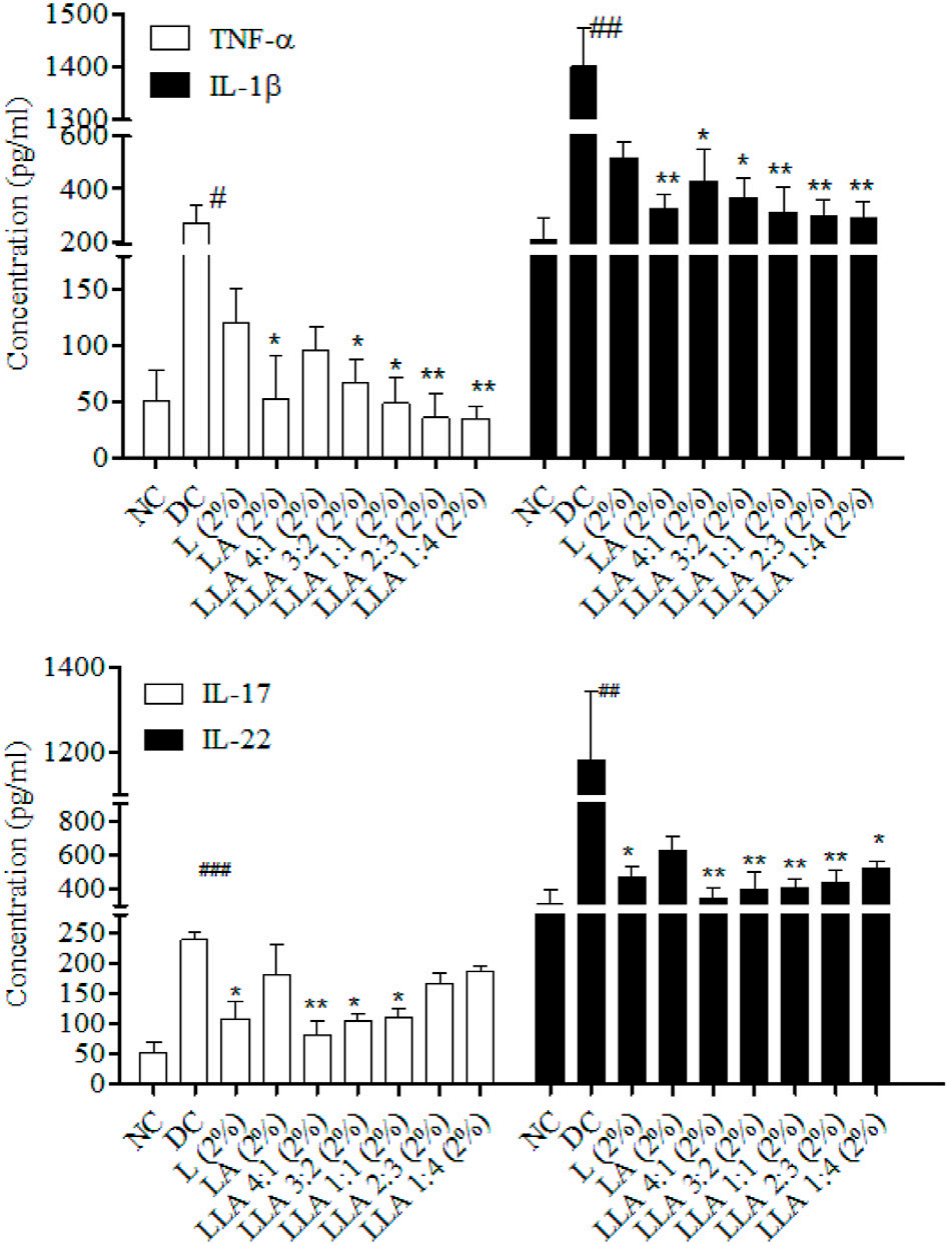
Levels of Th-17 and Th-1 cell-specific cytokines in skin tissues of linalool and linalyl acetate combination-treated mice. NC; normal control, DC; disease control, LLA (2%); linalool + linalyl acetate in 4:1, 3:2, 1:1, 2:3, and 1:4 ratios at 2% concentration. The data represent mean ± SEM, *n* = 6. ^#^ *p* < 0.05, ^##^ *p* < 0.01 and ^###^ *p* < 0.001 (NC vs. DC), *p < 0.05 and **p < 0.01 (DC vs. treatments).

A significant increase in TNF-α (*p* < 0.05) and IL-1β (*p* < 0.05) levels was observed in the DC group. The levels of IL-17 (*p* < 0.001) and IL-22 (*p* < 0.01) also increased significantly in psoriatic mice. LLA (2%) ratios 4:1, 3:2, and 1:1 showed significant alleviation in the level of IL-17. However, ratios 4:1, 3:2, 1:1, and 2:3 showed considerable alleviation in the level of IL-22. LLA (2%) ratios 3:2, 1:1, 2:3, and 1:4 showed significant alleviation in the levels of TNF-α, and ratios 4:1, 3:2, 1:1, 2:3, and 1:4 showed significant alleviation in the levels of IL-1β. LLA 2% (1:1 w/w) showed a consistent and better recovery in all these parameters among all combinations (Figure 3).

#### 3.1.2 Histological and immune histological changes

**FIGURE 4:**
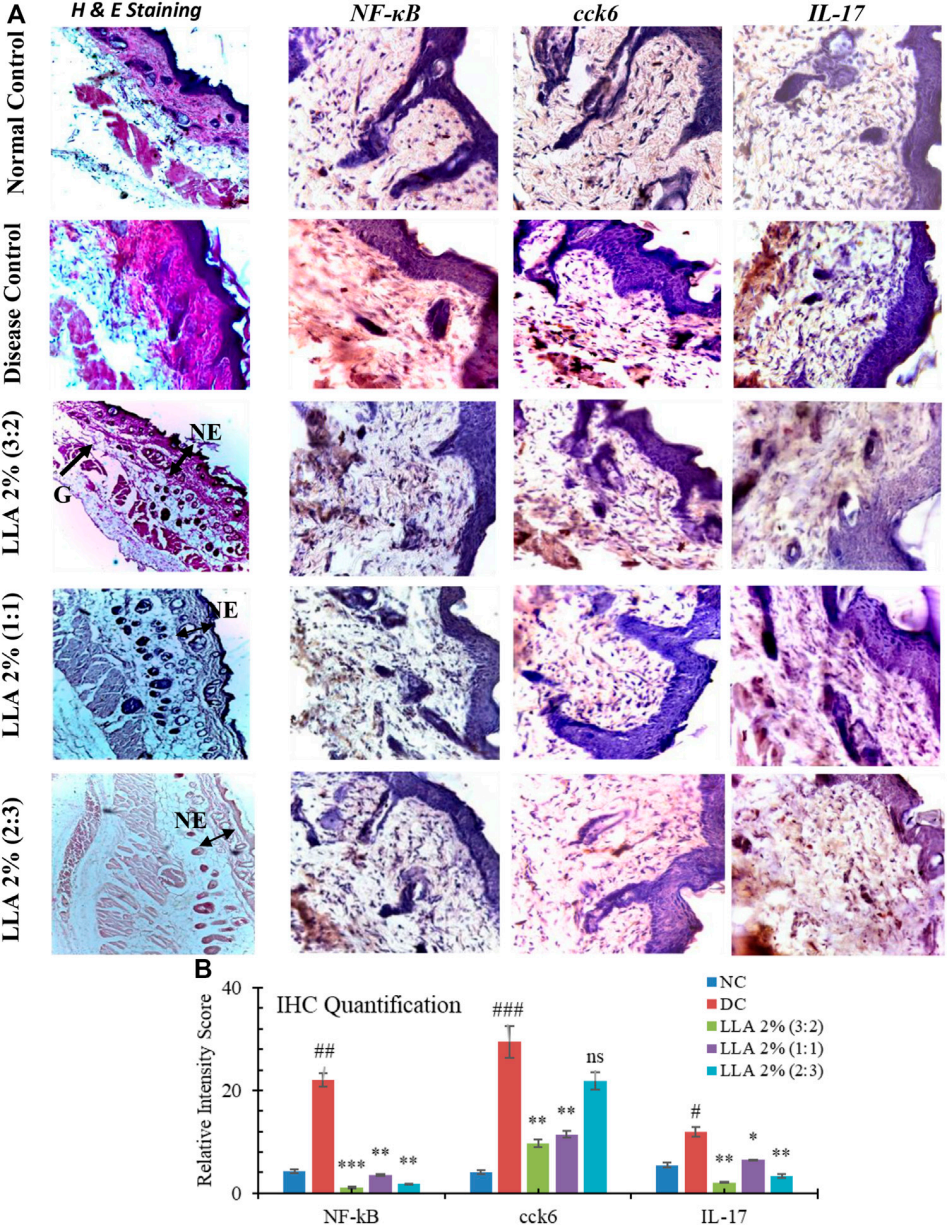
Histopathological features of the skin treated with linalool and linalyl acetate in combination. Immunohistochemical images of the mice skin treated with linalool + linalyl acetate combinations. **(A)** Histopathological features and immunohistochemical images of the mouse skin treated with linalool + linalyl acetate combinations. Sections were stained with H and E, and pictures were visualized at 10X magnification. **(B)** depicts the individual protein’s relative intensity scores of the IHC expressions. LLA (2%); linalool + linalyl acetate in 3:2, 1:1, and 2:3 ratios at 2% concentration. NE; normal epidermis, G; granulosis. The brown color represents NF-κB, IL-17, and cck6 expression.

Histological investigation depicts the significant presence of psoriasis-like histological changes in the psoriatic mice. These changes got normalized after the topical application of the test samples. The histological changes are summarized in Table 2; Figure 4. LLA 2.0% (1:1 w/w) showed an apparent reduction in keratinocyte proliferation, epidermal hyperplasia, and vacuole formation; hence, there were no erythema, flakiness, and skin thickness observed (very near to normal skin). An increase in the expressions of NF-κB, IL-17, and cck6 was observed in the DC group. LLA 2.0% (3:2 and 1:1 w/w) showed a marked reduction in the expression of NF-κB, IL-17, and cck6 (Figure 4). Quantification of IHC staining differentiated the individual group. As depicted in Figure 4B, the DC group shows significantly higher expression of the particular gene (*p* < 0.05). Except for LLA 2% (2:3), all the tested groups showed significant recovery in the expressions of cck6, and IL-17. LLA 2% (3:2) and LLA 2% (1:1) showed the best intensity recovery compared to the DC group.

**TABLE 2:** Histopathological features of linalool and linalyl acetate treated skin.

Group	Histopathological assessment	Remarks
Normal control	Normal epithelium and no vacuole	Normal architecture
Disease control	Higher keratinocytes, epidermal hyperplasia, and vacuole formation	Very thick skin, well-defined erythema, and flaky skin
LLA 2.0% (4:1 w/w)	Reduction in keratinocyte proliferation, epidermal hyperplasia, and vacuole formation	Less erythema, less flakiness, and no thickness
LLA 2.0% (3:2 w/w)	Reduction in keratinocyte proliferation, epidermal hyperplasia, and vacuole formation	No erythema, no flakiness, and thickness
LLA 2.0% (1:1 w/w)	Reduction in keratinocyte proliferation, epidermal hyperplasia, and vacuole formation	No erythema, no flakiness, and thickness, very near to normal skin
LLA 2.0% (2:3 w/w)	Reduction in keratinocyte proliferation, epidermal hyperplasia, and vacuole formation	No erythema, less flakiness, and thickness
LLA 2.0% (1:4 w/w)	Marginal changes	No erythema, flakiness, and thickness

LLA, linalool + linalyl acetate combination.

### 3.2 Acute dermal toxicity profile of LLA combination

Primary irritation indices of all the tested groups were found to be in the category of irritation barely perceptible. No significant change in the body weight of any tested animals, whether mice or rats, was observed. Animals in the gross pathological study showed no differences in the organs studied, including their absolute and relative weight (Tables 3, 4; Supplementary Figures S2, S3).

**TABLE 3:** PII, body, and organ weight of the mice topically treated with linalool + linalyl acetate combinations.

	Female				Male			
Parameter	Vehicle	LLA2%	LLA10%	LLA20%	Vehicle	LLA2%	LLA10%	LLA20%
PII	IBP	IBP	IBP	IBP	IBP	IBP	IBP	IBP
BW(g)	31.89 ± 01.25	32.39 ± 00.74	31.70 ± 01.62	31.33 ± 02.03	42.97 ± 01.27	41.47 ± 01.08	42.41 ± 02.40	41.06 ± 01.54
Brain(g)	00.40 ± 00.01	00.35 ± 00.05	00.39 ± 00.02	00.38 ± 00.02	00.46 ± 00.03	00.35 ± 00.03	00.39 ± 00.02	00.39 ± 00.09
Heart(g)	00.18 ± 00.01	00.18 ± 00.03	00.16 ± 00.02	00.19 ± 00.01	00.19 ± 00.02	00.19 ± 00.01	00.16 ± 00.02	00.18 ± 00.01
Lung (g)	00.17 ± 00.01	00.19 ± 00.01	00.21 ± 00.00	00.18 ± 00.01	00.19 ± 00.01	00.20 ± 00.01	00.21 ± 00.00	00.18 ± 00.01
Liver (g)	01.55 ± 00.02	01.62 ± 00.13	01.50 ± 00.10	01.73 ± 00.09	01.7 ± 00.02	01.69 ± 00.07	01.50 ± 00.06	01.63 ± 00.09
Kidney (g)	00.33 ± 00.02	00.34 ± 00.02	00.38 ± 00.02	00.32 ± 00.07	00.41 ± 00.01	00.34 ± 00.02	00.38 ± 00.02	00.31 ± 00.01
Spleen (g)	00.25 ± 00.02	00.21 ± 00.01	00.25 ± 00.05	00.24 ± 00.07	00.28 ± 00.02	00.31 ± 00.03	00.28 ± 00.06	00.24 ± 00.03

PII; primary irritation index, IBP; irritation barely perceptible, BW; body weight, LLA; linalool + linalyl acetate combination. Data represent mean ± SEM, n = 6.

**TABLE 4:** PII and body weight of the rats topically treated with linalool + linalyl acetate combinations.

Parameter	Vehicle	LLA2%	LLA10%	LLA20%	Vehicle	LLA2%	LLA10%	LLA20%
PII	IBP	IBP	IBP	IBP	IBP	IBP	IBP	IBP
BW (g)	244.80 ± 12.98	242.60 ± 18.21	241.90 ± 19.21	243.90 ± 19.14	292.30 ± 33.80	310.50 ± 26.35	306.40 ± 24.83	309.90 ± 20.46

PII, primary irritation index; IBP, irritation barely perceptible; BW, body weight; LLA, linalool + linalyl acetate combination. Data represent mean ± SEM, *n* = 6.

### 3.3 Repetitive dose dermal safety of LLA combination

Animals in the gross pathological study showed no changes in any of the organs studied, including their absolute and relative weights (Table 5). No observational changes in skin, morbidity, and mortality were observed throughout the experimental period in all the groups of the animals, even on the highest dose level of LLA (4%; 1:1 w/w ratio).

**TABLE 5:** Body and organ weight of the rats repeatedly treated with linalool + linalyl acetate combinations for 28 days, following topical administration.

Parameter		Female				Male			
		Vehicle	LLA0.2%	LLA2.0%	LLA4.0%	Vehicle	LLA0.2%	LLA2.0%	LLA4.0%
BW (g) at different days	1	240.67 ± 9.45	231.33 ± 16.50	217.33 ± 11.67	220.33 ± 14.01	378.33 ± 23.54	364.33 ± 30.92	381.00 ± 8.72	361.33 ± 22.90
	7	238.33 ± 14.36	233.33 ± 15.50	217.33 ± 12.86	219.00 ± 10.81	383.33 ± 25.70	365.67 ± 29.87	381.67 ± 9.29	357.00 ± 19.97
	14	240.33 ± 16.74	228.33 ± 25.66	221.33 ± 11.15	224.00 ± 5.19	385.67 ± 25.03	365.33 ± 29.57	378.00 ± 6.93	363.67 ± 15.82
	21	238.33 ± 18.47	231.67 ± 22.19	222.67 ± 10.07	227.00 ± 6.56	384.33 ± 20.74	360.00 ± 30.20	377.67 ± 11.93	360.33 ± 17.90
	28	236.00 ± 21.38	232.33 ± 16.56	219.67 ± 11.59	226.00 ± 3.61	384.00 ± 27.50	362.67 ± 32.32	382.33 ± 11.06	369.33 ± 11.02
Heart (g)		1.00 ± 0.03	1.07 ± 0.21	0.92 ± 0.06	1.04 ± 0.05	1.51 ± 0.18	1.30 ± 0.31	1.25 ± 0.21	1.38 ± 0.23
Lung (g)		2.81 ± 0.15	2.62 ± 0.49	2.64 ± 0.35	3.31 ± 0.27	5.60 ± 0.33	5.04 ± 1.45	3.93 ± 0.21	4.81 ± 0.65
Liver (g)		8.94 ± 0.07	8.98 ± 0.53	8.75 ± 0.23	8.81 ± 0.17	12.88 ± 1.97	11.04 ± 1.16	12.48 ± 0.48	12.41 ± 0.73
Kidney (g)		1.64 ± 0.10	1.82 ± 0.08	1.65 ± 0.07	1.86 ± 0.11	2.70 ± 0.14	2.17 ± 0.05	2.46 ± 0.04	2.34 ± 0.33
Spleen (g)		0.84 ± 0.07	0.94 ± 0.07	0.80 ± 0.05	0.96 ± 0.03	1.02 ± 0.03	1.07 ± 0.03	0.99 ± 0.16	1.03 ± 0.09

BW, body weight; LLA, linalool + linalyl acetate combination. Data represent mean ± SEM, *n* = 6.

The blood and serum analysis showed non-significant changes in all the studied parameters like hemoglobin level, RBC count, WBC count, differential leukocyte count, SGPT, SGOT, serum protein, triglycerides, cholesterol, and albumin, except serum creatinine and ALP (Table 6; Supplementary Figure S4).

**TABLE 6:** Effect of LLA (1:1 w/w ratio) at 0.2%, 2%, and 4% PEG200 solutions on different biochemical and hematological parameters when applied topically in Wistar rats once-a-day for 28 days..

Parameter	Group			
	Control	0.2%	2.0%	4.0%
Hemoglobin (g/dL)	19.79 ± 1.69	20.40 ± 1.56	20.97 ± 0.90	23.20 ± 2.32
Albumin (g/dL)	2.27 ± 0.53	2.22 ± 0.40	2.41 ± 0.38	2.60 ± 0.28
Serum protein (mg/ml)	3.36 ± 0.54	2.91 ± 0.12	3.18 ± 0.47	3.25 ± 0.49
Creatinine (mg/dL)	0.82 ± 0.07	0.82 ± 0.01	0.84 ± 0.07	0.83 ± 0.05
Bilirubin (mg/dL)	0.45 ± 0.03	0.42 ± 0.03	0.45 ± 0.03	0.41 ± 0.10
Cholesterol (mg/dL)	0.09 ± 0.01	0.08 ± 0.01	0.09 ± 0.01	0.07 ± 0.01
Triglycerides (mg/dL)	130.04 ± 11.92	134.40 ± 20.59	133.70 ± 11.82	97.14 ± 15.51
ALKP (U/L)	375.76 ± 41.30	329.87 ± 15.08	249.84 ± 22.38*	168.84 ± 10.98*
SGOT (U/L)	29.61 ± 5.96	25.44 ± 2.66	30.26 ± 7.50	44.00 ± 7.55
SGPT (U/L)	33.47 ± 1.04	31.32 ± 1.99	31.81 ± 3.56	34.00 ± 3.55
RBC (millions/mm^3^)	08.85 ± 1.22	08.23 ± 0.40	06.70 ± 0.90	07.86 ± 0.74
WBC (thousands/mm^3^)	31.33 ± 9.78	33.81 ± 8.39	19.03 ± 4.11	20.98 ± 1.22
Differential leucocyte count				
Lymphocytes	71.50 ± 6.03	51.00 ± 4.27	53.75 ± 8.11	66.75 ± 7.76
Neutrophils	10.16 ± 1.48	9.75 ± 3.06	09.75 ± 2.84	10.50 ± 3.50
Monocytes	12.50 ± 2.63	17.00 ± 3.31	24.75 ± 5.17	16.75 ± 2.60
Eosinophils	06.25 ± 0.93	01.25 ± 0.69	02.75 ± 0.72	4.751 ± 0.32

BW, body weight; LLA, linalool + linalyl acetate combination. Data represent mean ± SEM, *n* = 6.

### 3.4 Stability of linalool and linalyl acetate combination

**FIGURE 5:**
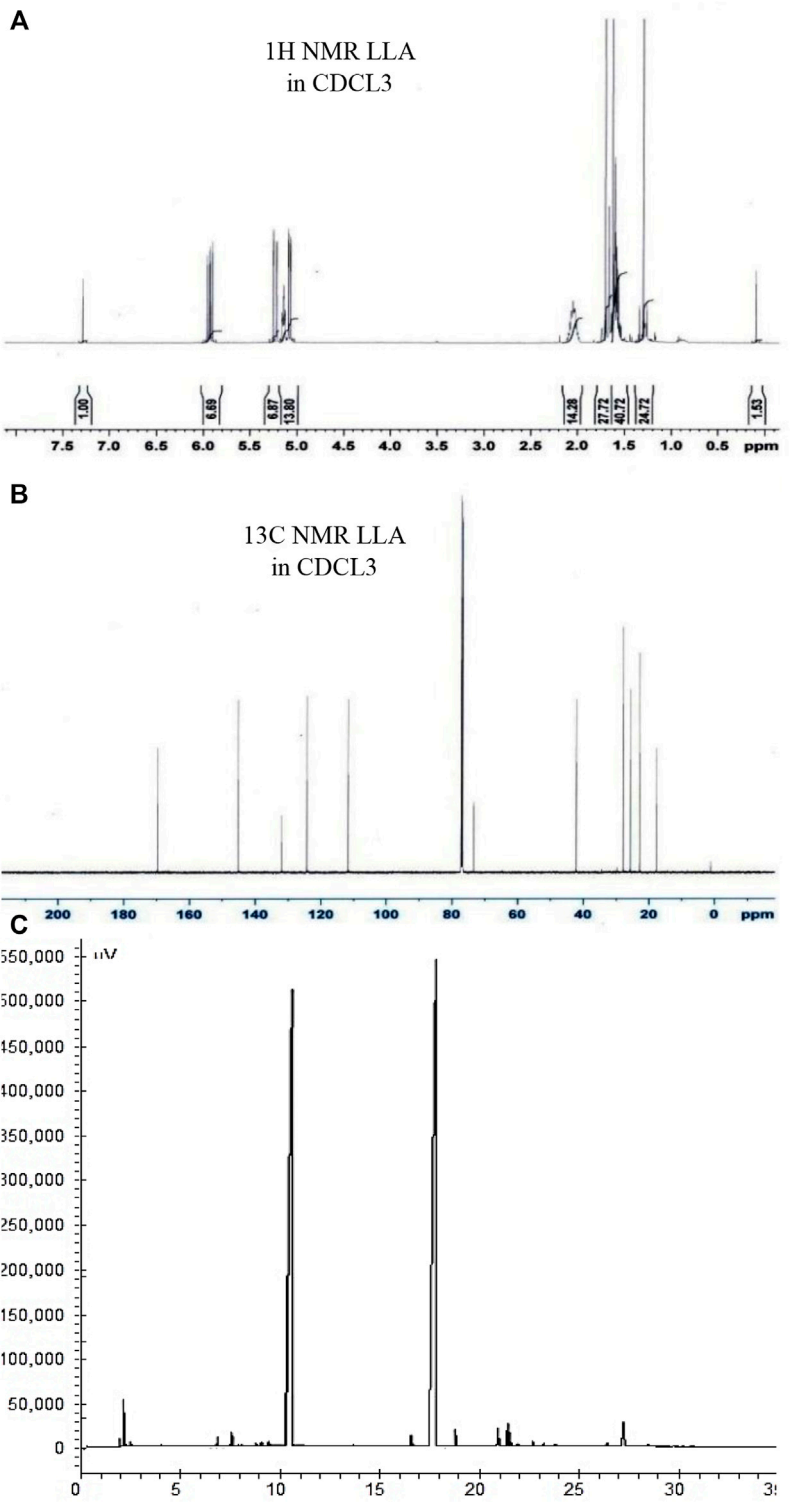
Characterizing the phyto-combination of linalool and linalyl acetate by NMR and GC analysis. Pictures **(A)**, **(B)**, and **(C)** depict ^1^H NMR, ^13^C NMR spectrum, and GC chromatogram of linalool + linalyl acetate combination (1:1 w/w), respectively.

Linalool and linalyl acetate in 1:1 w/w ratio were analyzed by gas chromatography to confirm their intactness in combination. The purity of linalool and linalyl acetate was found to be 49.12% and 49.56%, respectively (Figure 5). Linalool and linalyl acetate peaks in 1D ^1^H NMR spectra are ascribed to the protons at R_2_CH_2_, C = C-CH_3_, R_3_OH, R_2_C = CHR, and RCH = CH_2_. The observed peaks in the 1D^13^C NMR spectra were found to be in the four central regions that were ascribed to the presence of different types of carbon, such as aliphatic, allylic, hydroxy, and vinylic groups (Figure 5).

## 4 Discussion

In our previous study, we reported the effectiveness of lavender oil (LO) against psoriasis. Linalool, linalyl acetate, and lavandulol were observed as the main components of LO. The most active components against psoriasis were linalool and linalyl acetate. Linalool showed more than 50% recovery in PASI scores and primarily alleviated the level of Th-17 cell cytokines. Linalyl acetate showed the efficient recovery of Th-1 cytokines (94%). Unlike linalyl acetate, linalool showed better improvement in skin thickness as observed from the histological changes in the psoriatic skin (Rai et al., 2020). As these molecules worked independently on different targets to mitigate psoriasis-like conditions, the synergy between L and LA in treating IMQ-induced psoriasis was explored. Therefore, the synergistic or additive effect of L and LA against psoriasis and their safety in the long-term topical application were investigated thoroughly.

PASI and CosCam scores portray the development of the psoriasis-like symptoms. This investigation was performed to have an insight into the induction of the disease and the pattern of cure (Van Der Fits et al., 2009). PASI and CosCam scorings were done based on the phenotypic assessment criteria of the skin (Žurauskas et al., 2020). Reported literature suggests that more than 50% recovery in PASI scores confirms a considerable improvement in psoriasis (Carlin et al., 2004), and more than 75% recovery in PASI is a significant improvement in psoriatic conditions with immense utility (Abrouk et al., 2017). As per these criteria, only LLA 2% (1:1 w/w) could attain PASI 75, and the same was supported by CosCam analysis also. PASI 50 was achieved by all the ratios selected for the investigation. CosCam pictorial and radar graphs show the relevance of different proportions of LLA 2% in psoriasis (Figure 2).

Different types of cytokines (i.e., Th-17 specific and Th-1 specific) are released in IMQ-induced psoriasis-like conditions (Van Der Fits et al., 2009). Attainment of PASI 75 and a significant reduction in cytokine levels are always appreciated. A high percent recovery in the hyper-expressed cytokines was observed. As per our previous report, linalool reduces the level of Th-17 cells, and linalyl acetate reduces Th-1 cells specific cytokines efficiently (Li et al., 2018). However, in combination, these molecules have shown excellent recovery in both types of cytokines. As shown in Table 1, the combination of L and LA in a 1:1 ratio shows a higher reduction (denoted in terms of % recovery towards normalization) in the PASI score, CosCam score, levels of TNF-α, IL-1β, IL-17, and IL-22 when compared with the individual effect of L and LA. In such conditions, where the total dose of drugs in combination is not more than a dose of any particular drug, while the combined pharmacological effect is better than any individual drug used in the treatment, the result is said to be synergistic (Yadav et al., 2008; Chambers (2001)). As per the Huo et al. (2013) report, L and LA inhibit the NF-κB and MAPK activation and reduced the level of IL-6 and TNF-α (Huo et al., 2013). Results of this investigation were found to concur with the reduction in the level of these psoriasis-specific cytokines treated with terpenes and terpenoids (Li et al., 2018).

Thickened skin in psoriatic conditions occurred due to the keratinocyte’s hyper-proliferation and redness/erythema due to the induction of angiogenesis in response to the antigen. These changes led to the exacerbation of psoriasis, and when the condition worsens, the disease gets more severe (Goedkoop et al., 2004). The imbalanced innate and adaptive immune responses initiate psoriatic conditions where IL-1β, IL-6, IL-8, IL-12, IFN-γ, and TNF-α get expressed (Asadullah et al., 1999). In IMQ-induced psoriasis, TLR-7/8 gets activated and initiates Th-17 cells specific cytokines in response to the IL-23 overproduction by plasmacytoid dendritic cells. IL-23 binds with the CD4^+^ cells available in lymph nodes locally. Matured CD4^+^ cells are then converted to pathogenic Th-17 cells and produce IL-17 and IL-22 (Van Der Fits et al., 2009; Sharma et al., 2022). IL-17 and IL-22 expressions initiate keratinocyte hyperproliferation and down-regulation of genes associated with keratinocyte differentiation. According to Furue et al., 2020, keratinocytes produce an array of antimicrobial peptides and cytochemokines in response to the produced IL-17 A that further exacerbates the psoriasis like inflammatory condition. The release of IL-17 is directly linked to the worsening of the keratinocyte proliferation, that could be alleviated by reducing IL-17 level (Furue et al., 2020). Our results were in reasonable agreement with this finding, where alleviation in the proliferation of the keratinocytes was observed with the reduction in the level of Th-17 cells specific cytokines.

Literature reports epidermal and dermal hyperplasia, parakeratosis, hyperkeratosis, dermal edema, vesicular formation, and granulosis in the psoriatic skin (Morsy et al., 2010). The significant histological changes were the epidermal thickening due to hyperkeratosis, parakeratosis, dermal hyperplasia, granulosis, para-keratosis, intercellular edema, and vesicular formation in psoriatic mice (Tirumalae, 2013; Huang et al., 2019). Considerable reduction in these histological changes was observed in the case of LLA-treated groups, and the best result was observed in the case of a 1:1 w/w ratio of LLA 2%. Histological improvement confirms the anti-hyperproliferative effect of the tested combination. There is a possibility that linalool is impeding the process of proliferation as some of the investigations cite its anti-proliferative effect as well (Popadic et al., 2008).

Monocytes, macrophages, and plasmacytoid dendritic cells usually work as Antigen-Presenting Cells (APCs) and begin to express 7th or 8th type TLRs in response to IMQ or other stimuli. However, these APCs accept responses only from specific stimuli. APCs maturation initiates type 1 IFN activity owing to which the rapid influx of various immune cells takes place at the prone site. Van der Fits *et al.* report that, after pDC activation, further infiltration and influx of pDC takes place that facilitates many inflammatory responses. The initiation of IL23/Th-17 cascade after pDC infiltration and type I IFN activity is the most important hallmark of psoriasis-like conditions (Sharma et al., 2022). NF-κB is triggered in response to the TLR8 activation by IMQ. This activation leads to IL-23 and IL-6 releases. The binding of IL-23 with the receptors present on γδ T cells (cck6) triggers the differentiation of cck6 and hence, initiates the release of IL-17 and IL-22 (Sun et al., 2013). Based on this pathway, NF-κB, IL-17, and cck6 were checked for hyper-expression in skin tissue of different treatment groups (Kim et al., 2020). LLA 2% showed a marked alleviation in the expression of the selected markers in a 1:1 ratio. This may be due to the competitive binding of LLA with the TLR8 resulting in decreased phosphorylation of Ik-β in the cytosol (Somagoni et al., 2015; Li et al., 2018). L and LA are chemically terpenes and isolated from aromatic oils. Interestingly hispidulin, which is an aromatic oil component, significantly alleviates the imiquimod-induced psoriasis-like skin inflammation by inhibiting splenic Th1/Th17 cell population and keratinocyte activation. As per our results, the best possible mechanism that L and LA are following in combination could be the inhibition of Th1/Th17 cell population. Marked reduction in epidermal thickness, inflammation, and the expression of selected proteins (NF-κB, IL-17, and cck6) proves LLA’s immense utility in managing psoriasis. It supports the findings of Huo et al. (2013) that state the blocking of NF-kB activation is the linalool’s major mechanism of action.

**FIGURE 6:**
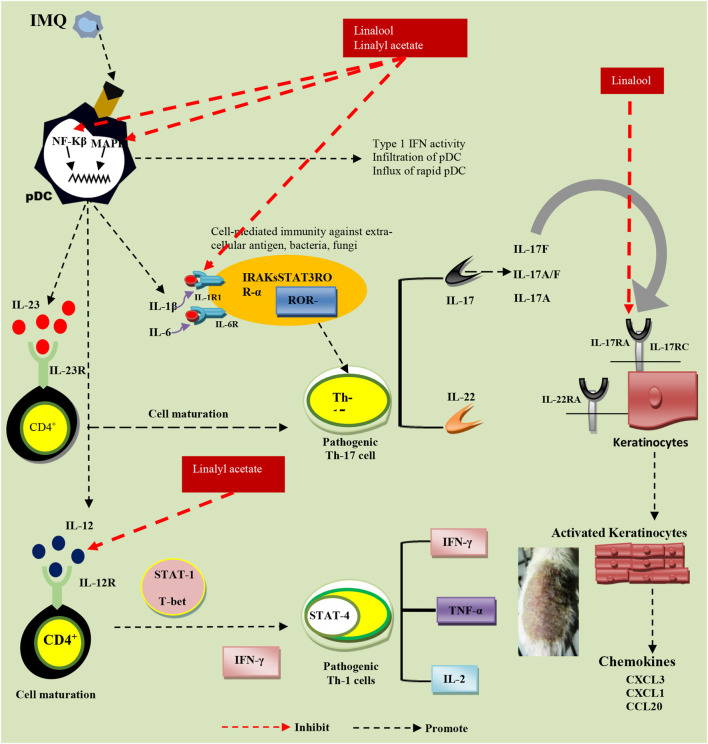
Probable pathways of linalool and linalyl acetate effectiveness against IMQ-induced psoriasis.

As linalool is primarily alleviating Th-17 cell cytokines and reducing skin thickness, it is expected to interfere with the process of activation of NF-kB and binding of IL-17 subset cytokines such as IL-17 A and IL-17 F with their respective receptors present on keratinocytes (the major reason behind the severe keratinocyte proliferation). There are two possibilities that, linalool is modifying both immune responses as well as the process of keratinocyte proliferation. At the same time, linalyl acetate seems to interfere with the IL-12-driven induction of the inflammatory responses owing to which it is working efficiently on Th-1 cells specific cytokines majorly (Figure 6). Mitigating Th-1 and Th-17 cells cytokines specific pathways has been identified as the best approach against IMQ-induced psoriasis (Tokura et al., 2010; Benham et al., 2013; Varma et al., 2017; Moos et al., 2019; Li et al., 2020). Therefore, exploring a combination of L and LA for their synergy in treating IMQ-induced psoriasis was attempted. Here, finding a ratio that shows the best result in the inhibition of the Th1/Th17 cell population is of prime importance.

Skin irritation is barely perceptible at the therapeutic dose, ensuring its high safety. No change in the mice’s body and organ weight and the rat’s body weight ensure the tested combination’s safety if it is available in systemic circulation to any extent (Han et al., 2012).

The period of psoriasis treatment is usually long and requires repeated topical administration of the therapeutic dose for a long duration. In long-term use, the administered dose could have adverse effects. However, LLA 2% (1:1 ratio) did not show any sign of toxicity. No change in the absolute and relative weights of the animal’s body and organ weights and nominal observational changes in skin ensure a good safety profile of the tested combination. It has shown no morbidity and mortality in all the experimental animals, even at the highest dose level of LLA (4%; 1:1 w/w ratio). It ensures its safety in long-term use whether this drug combination is available in systemic circulation or not following topical administration. The systemic availability of this combination could also lead to a change in the blood and hematological parameters (Surekha et al., 2012). However, we did not observe any change in the blood parameters even on LLA 4% dose compared to the vehicle-treated group.

The different combinations of linalool and linalyl acetate tested in this investigation were also checked for their compatibility in a mixture. GC chromatographic analysis confirms the molecules’ intactness when used in a 1:1 w/w ratio. NMR investigation showed the presence of all types of characteristic hydrogens and carbons peaks of linalool and linalyl acetate. This helped us to conclude that these molecules are mutually stable in combination.

## 5 Conclusion

The study investigated the combined effect of L and LA for the comprehensive management of psoriasis-like inflammation and its safety in long-term topical use. LLA 2% 1:1 w/w ratio was able to attain PASI 75 compared to the other tested ratios (achieved PASI 50) of L and LA. The combination shows synergistic activity because L and LA in combination (1:1 ratio) consistently exhibit higher anti-psoriatic activity (Table 1, denoted by percent recovery) when compared with either L or LA. This combination showed marked restoration in the histological and immune-histological changes towards normal compared to the psoriatic group. The mixture was found to be safe in single dosing and repeated dosing schedules. It proves the safety profile of the tested combination even in long-term use. Our previous work (Rai et al., 2020) was the scientific validation of the traditional knowledge that lavender oil and its constituents can be used to mitigate the psoriasis-like skin inflammation but the present work provides the novel fact that the linalool and linalyl acetate in combination exhibit the synergistic effect against psoriasis. This fact helped us to file the Indian Patent, which is under evaluation with the Indian Patent Office. Finally, we can conclude that the present study demonstrates the synergistic effect of LLA 2% 1:1 w/w ratio in attaining PASI 75 and provides strong scientific safety evidence favoring its topical use in humans.

## Data Availability

The original contributions presented in the study are included in the article/Supplementary Material; further inquiries can be directed to the corresponding author.

## Abbreviations

cck6, chemokine receptor 6; CD4^+^, cluster of differentiation 4; DAB, 3,3-diaminobenzidine; DC, disease control; ELISA, enzyme-linked immunosorbent assay; GC, gas chromatography; i.p., intra-peritoneal; IFN-γ, interferon-gamma; IL, interleukin; IMQ, imiquimod; L, linalool; LA, linalyl acetate; LPS, lipopolysaccharide; MAPK, mitogen-activated protein kinase; NC, normal control; NF-κB, nuclear factor kappa-light-chain-enhancer of an activated B cell; OECD, Organization for Economic Co-operation and Development; PASI, psoriasis area and severity index; pDCs, paracitoid dendritic cells; PEG, polyethylene glycol; PII, primary irritation index; Th-1, 1st type helper T cell; Th-17, 17th type helper T cell; Th-22, 22nd type helper T cell; TLR, toll-like receptor; TNF, tumor necrosis factor.
