# Supplementary material for: A combination of linalool and linalyl acetate synergistically alleviates imiquimod-induced psoriasis-like skin inflammation in BALB/c mice
Source: Front Pharmacol. 2022 Aug 5;13:913174. doi: 10.3389/fphar.2022.913174 (PMC9388787; doi:10.3389/fphar.2022.913174)
Supplement: Supplementary file 1 [file Table1.DOCX]

Date: 5^th^ April 2022

To,

The Editor-in-Chief,

Frontiers in Pharmacology

**Subject:** Submission of ***research article*** for the purpose of publication in your esteemed journal.

Dear Sir,

I wish to submit a manuscript entitled “A combination of linalool and linalyl acetate synergistically alleviates imiquimod-induced psoriasis-like skin inflammation in BALB/c mice” in your esteemed journal.

The effect of *Lavandula angustifolia* Mill essential oil and its major phytoconstituents linalool (L) and linalyl acetate (LA) is well defined against psoriasis. Their distinctive affinity on the psoriasis targets suggested us to explore their combined effect against psoriasis. In this investigation, we checked the synergy between L and LA, if any, against psoriasis and their safety in long-term topical uses. The anti-psoriatic activity was done using IMQ induced psoriasis in BALB/c mice however the acute and repetitive dose dermal toxicity studies were carried out as per the OECD guidelines. This study proves the synergy between L and LA when used in 1:1 w/w ratio (2%) for the treatment of psoriasis like condition and provides strong scientific evidence for its safe topical use.

**With Best Regards**

Dr. Narayan Prasad Yadav

Principal Scientist

CSIR-Central Institute of Medicinal and Aromatic Plants,

P. O. CIMAP, Lucknow (U.P.) 226 015 India

Email: [np.yadav@cimap.res.in](mailto:np.yadav@cimap.res.in), [npyadav@gmail.com](mailto:npyadav@gmail.com),

Phone: +91-522-2718657, Fax: +91-522-2342666
