# Supplementary material for: A combination of linalool and linalyl acetate synergistically alleviates imiquimod-induced psoriasis-like skin inflammation in BALB/c mice
Source: Front Pharmacol. 2022 Aug 5;13:913174. doi: 10.3389/fphar.2022.913174 (PMC9388787; doi:10.3389/fphar.2022.913174)
Supplement: Supplementary file 2 [file Presentation1.PPTX]

## Slide 1
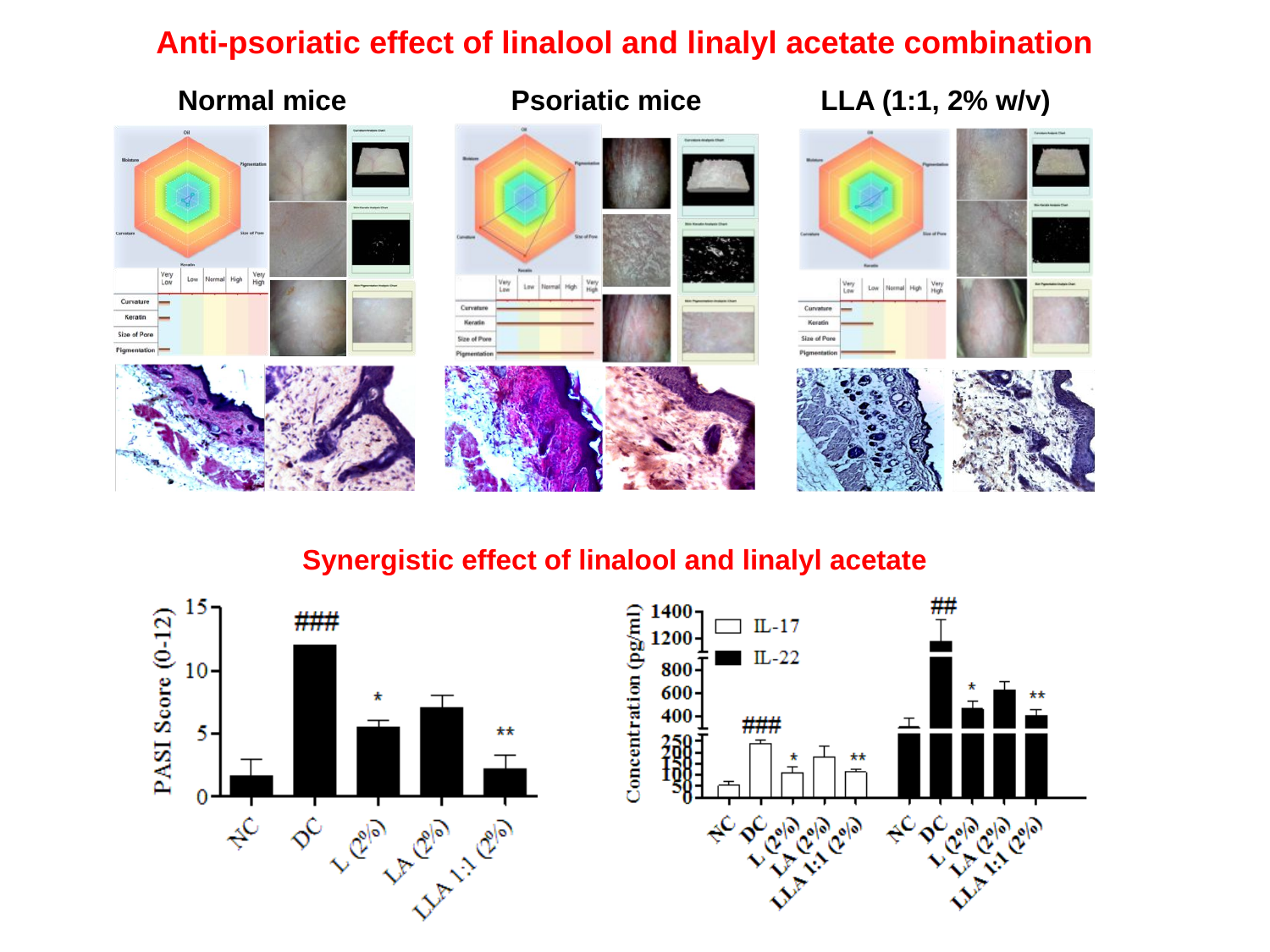

Anti-psoriatic effect of linalool and linalyl acetate combination
Normal mice
Psoriatic mice
LLA (1:1, 2% w/v)
Synergistic effect of linalool and linalyl acetate
