# Supplementary material for: A combination of linalool and linalyl acetate synergistically alleviates imiquimod-induced psoriasis-like skin inflammation in BALB/c mice
Source: Front Pharmacol. 2022 Aug 5;13:913174. doi: 10.3389/fphar.2022.913174 (PMC9388787; doi:10.3389/fphar.2022.913174)
Supplement: Supplementary file 3 [file Table2.DOCX]

***Credit Author statement***

Conceptualization, and conceiving of the idea, analysis of the collected data and manuscript writing of the original draft were done by ***NPY***. Conceiving the idea, experimentation, compilation of data and manuscript writing were done by ***VKR***. Experimentation and analysis of data of the toxicity studies were done by ***DC*** and instrumental analysis and data interpretation were done by ***CSC.*** The final draft of the manuscript was checked and approved from the all the authors before submission.
