# Supplementary material for: A combination of linalool and linalyl acetate synergistically alleviates imiquimod-induced psoriasis-like skin inflammation in BALB/c mice
Source: Front Pharmacol. 2022 Aug 5;13:913174. doi: 10.3389/fphar.2022.913174 (PMC9388787; doi:10.3389/fphar.2022.913174)

**Supplementary Material 1**

**Quality control of linalyl acetate and linalool**

Linalool (Catalogue number W263516) and Linalyl acetate (Catalogue number W263613) of natural origin were procured from Sigma-Aldrich. The product specifications sheet from Sigma-Aldrich is given below. The in house GC analysis was also performed for quality control of Linalool and Linalyl acetate (Fig. SM1)

**Figure SM1:** Purity profile of linalyl acetate and linalool


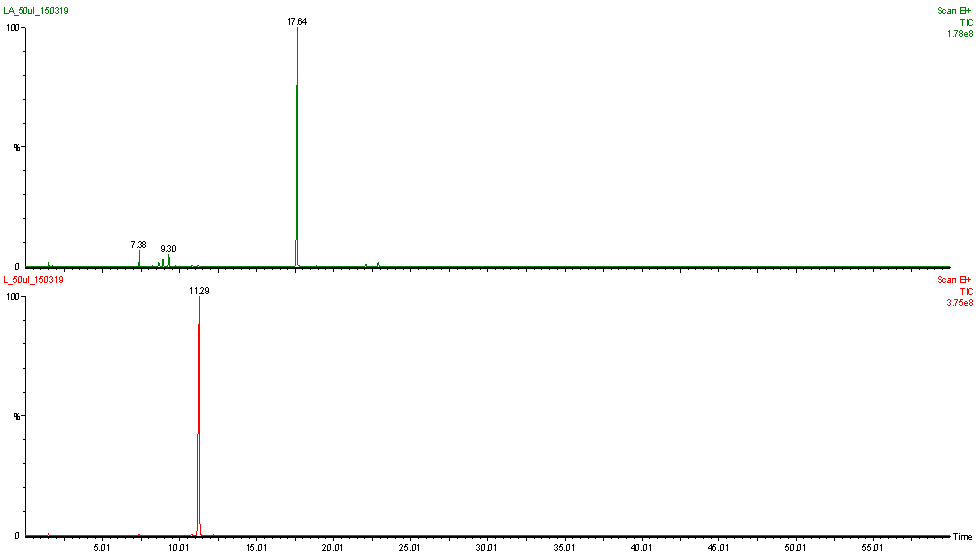


Linalool

Linalyl acetate

Purity ≥80 %

Purity ≥95%


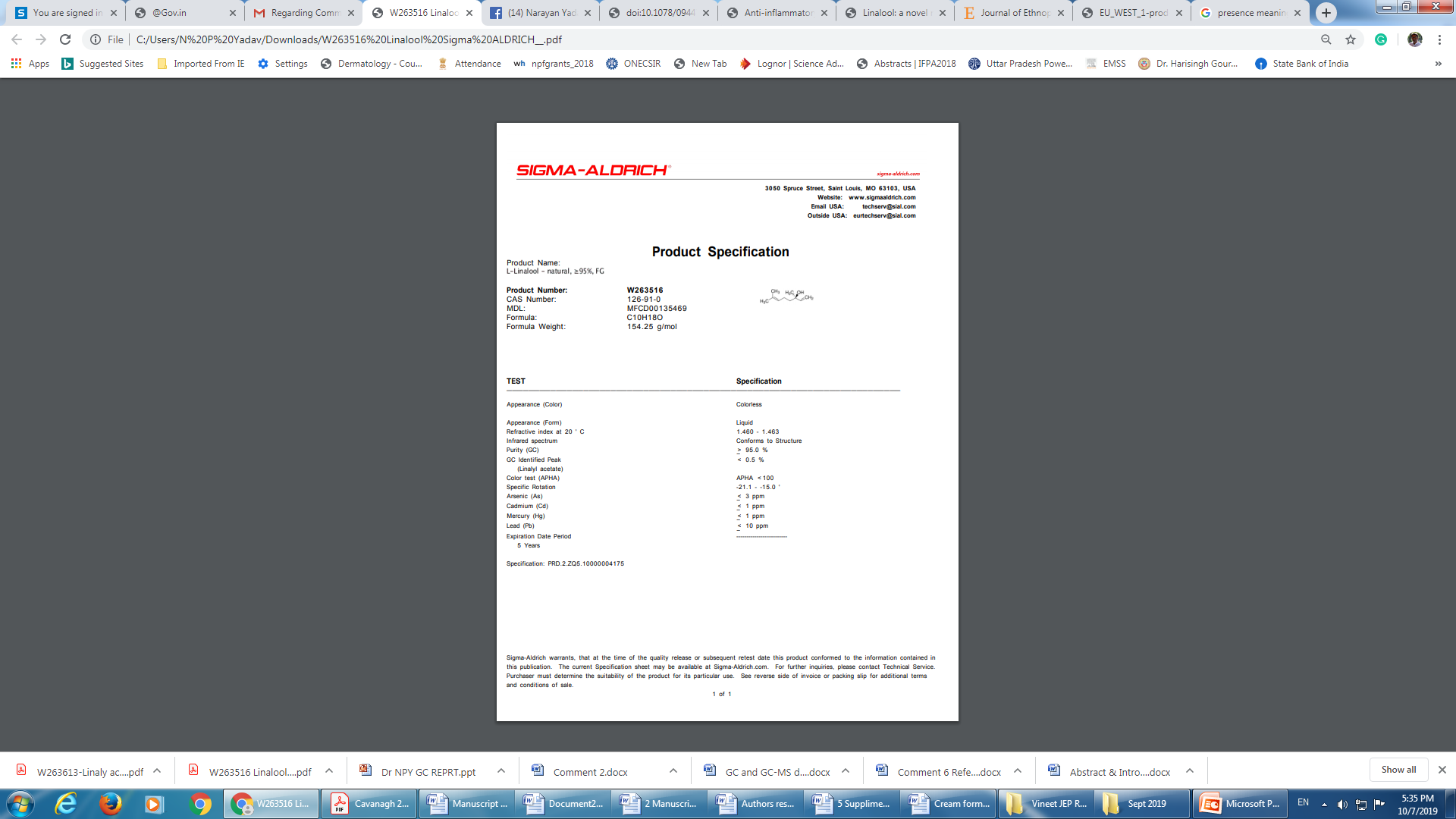


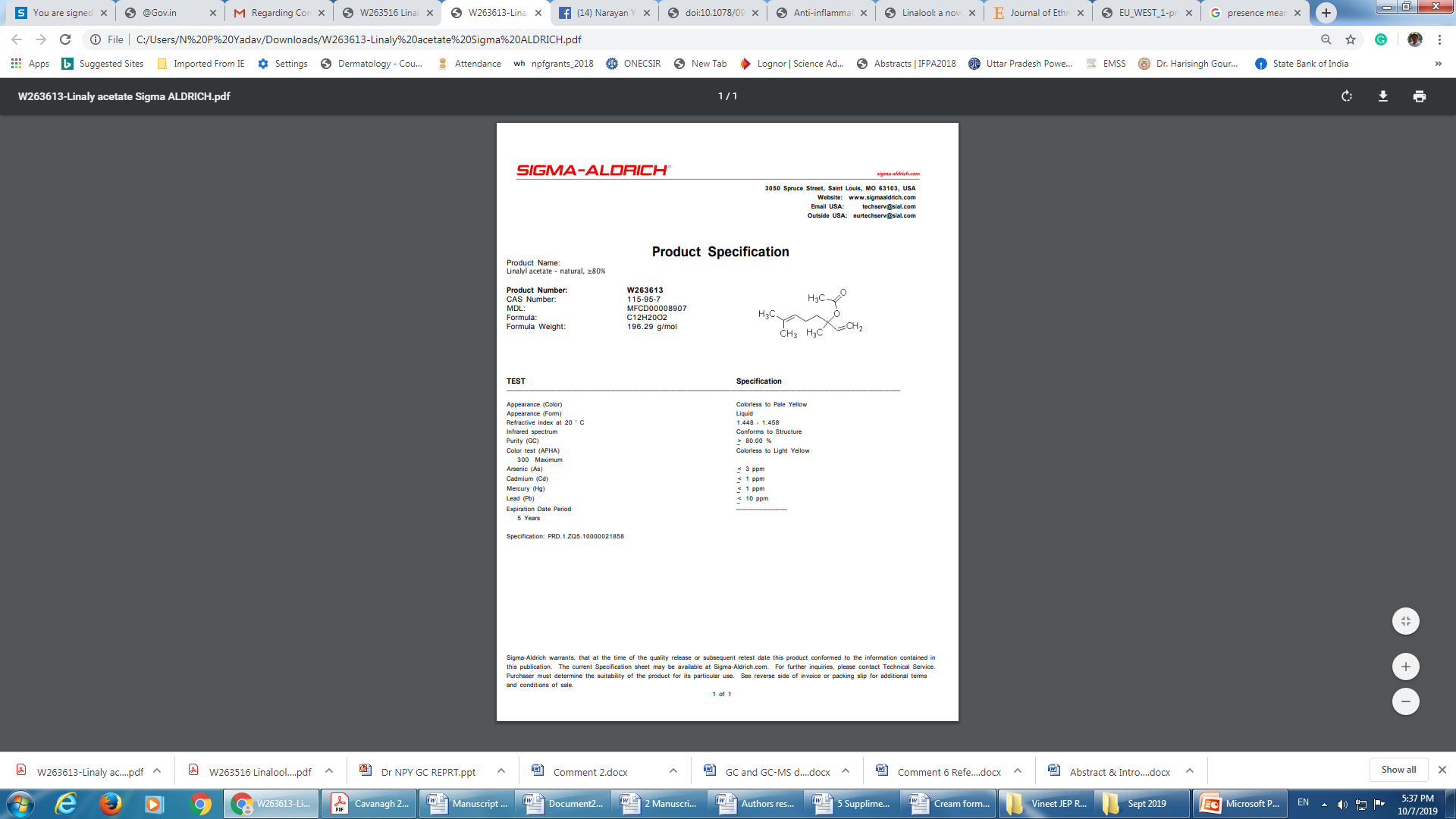

Supplement: Supplementary file 4 [file Table5.DOCX]
