# Supplementary material for: A combination of linalool and linalyl acetate synergistically alleviates imiquimod-induced psoriasis-like skin inflammation in BALB/c mice
Source: Front Pharmacol. 2022 Aug 5;13:913174. doi: 10.3389/fphar.2022.913174 (PMC9388787; doi:10.3389/fphar.2022.913174)
Supplement: Supplementary file 5 [file Table3.DOCX]

Date: 5th April 2022

To,

The Editor-in-Chief,

Frontiers in Pharmacology

**Subject:** Submission of ***research article*** for the purpose of publication in your esteemed journal.

Dear Sir,

It is stated that all authors have read and approved this version of the article and no part of this paper has been published nor it is simultaneously submitted elsewhere for publication.

The Indian patent (application number 201711031817) has been filed with the findings of this study, which is under review with the patent authority.

Experimental protocols for anti-inflammatory activity (AH-2012-05), acute dermal toxicity study (AH-2012-01), and repeated dose dermal toxicity study (AH-2012-01) were duly approved by the Institutional Animal Ethics Committee (400/01/AB/CPCSEA), Government of India. Animal experiments were conducted following the principles for laboratory animal use and care as found in European Community guidelines (EEC Directive of 1986; 86/609/EEC). The safety assessments were commenced in a single trial based on the Organization for Economic Cooperation and Development (OECD) guidelines 404 and 410.

The authors declare that there is no conflict of interest.

**With Best Regards**

Dr. Narayan Prasad Yadav

Principal Scientist

CSIR-Central Institute of Medicinal and Aromatic Plants,

P. O. CIMAP, Lucknow (U.P.) 226 015 India

Email: [np.yadav@cimap.res.in](mailto:np.yadav@cimap.res.in), [npyadav@gmail.com](mailto:npyadav@gmail.com),

Phone: +91-522-2718657, Fax: +91-522-2342666
