# Supplementary material for: A combination of linalool and linalyl acetate synergistically alleviates imiquimod-induced psoriasis-like skin inflammation in BALB/c mice
Source: Front Pharmacol. 2022 Aug 5;13:913174. doi: 10.3389/fphar.2022.913174 (PMC9388787; doi:10.3389/fphar.2022.913174)
Supplement: Supplementary file 6 [file DataSheet2.docx]

***Original research article***

A combination of linalool and linalyl acetate synergistically alleviates imiquimod-induced psoriasis-like skin inflammation in BALB/c mice

***Author names***

Vineet Kumar Rai^1^, Debabrata Chanda^1^, Chandan Singh Chanotiya^2^, Narayan Prasad Yadav^1*^

***Affiliations:***

^1^Bio-prospection and Product Development Division,

CSIR-Central Institute of Medicinal and Aromatic Plants, Lucknow, 226015, U.P., India.

^2^Phytochemistry Division,

CSIR-Central Institute of Medicinal and Aromatic Plants, Lucknow, 226015, U.P., India.

^*^**Correspondence:**

Dr. Narayan Prasad Yadav

Principal Scientist

Herbal Medicinal Product Lab, Bio-prospection and Product Development Division

CSIR-Central Institute of Medicinal and Aromatic Plants,

P. O. CIMAP, Lucknow (U.P.) 226 015 India

Email: [np.yadav@cimap.res.in](mailto:np.yadav@cimap.res.in), [npyadav@gmail.com](mailto:npyadav@gmail.com),

Phone: +91-522-2718657, Fax: +91-522-2342666

**Figure SM2:** Acute toxicity of linalool + linalyl acetate (2, 10 and 20%) in either sex of mice

**Figure SM3:** Acute toxicity of linalool + linalyl acetate (2, 10 and 20%) in either sex of rats

**Figure SM4:** Images of the female and male rats subjected for the sub-acute dermal toxicity study of LLA (1:1) at 0.2, 2.0 and 4.0 % dose.

**Figure SM2**

*Female mice Male mice*

Vehicle LLA2% LLA10% LLA20% Vehicle LLA2% LLA10% LLA20%


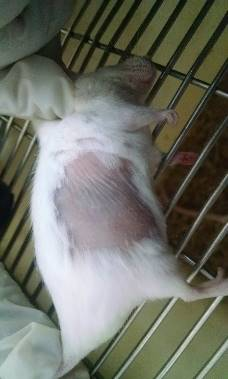

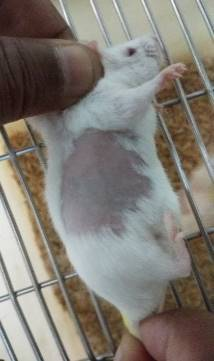

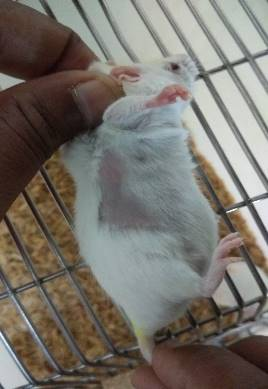

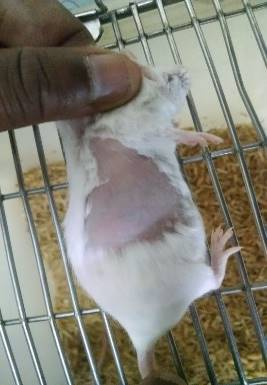

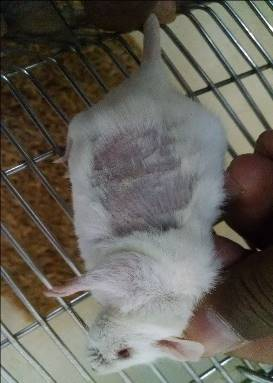

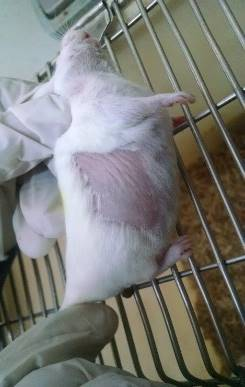

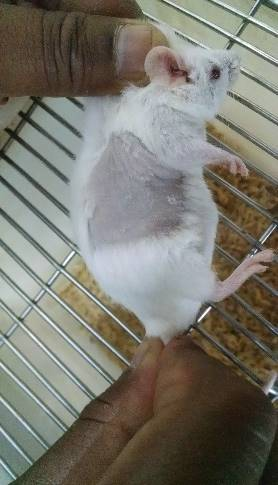

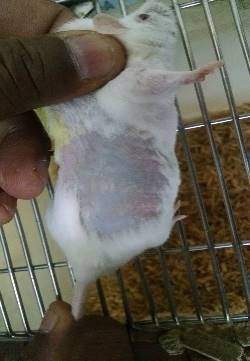


………………………….……………..Day 1^st^…….…………………………………


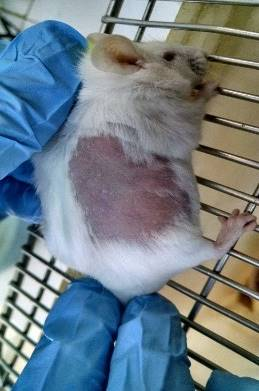

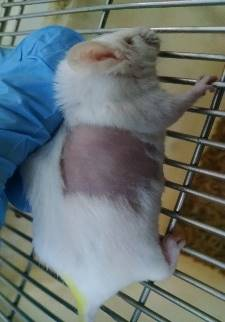

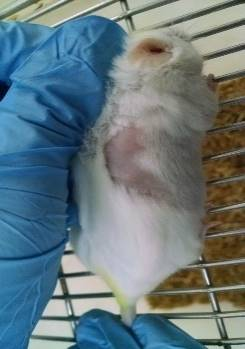

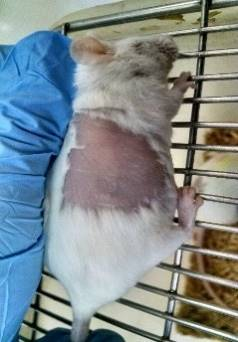

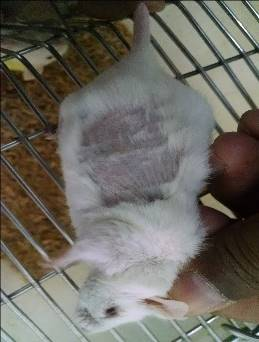

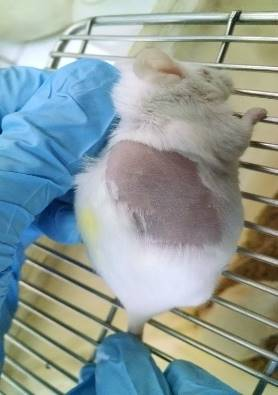

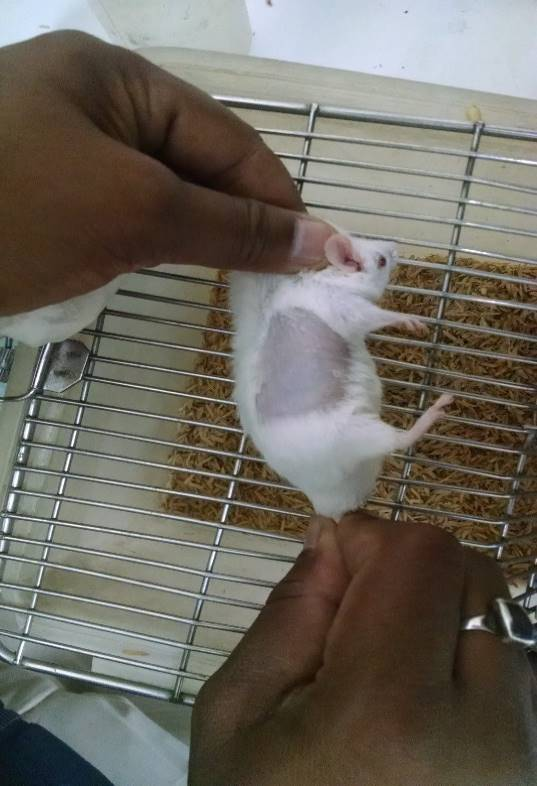

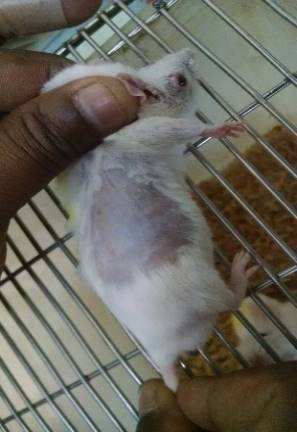


…………………………………….……..Day 7^th^…………..…………………………….


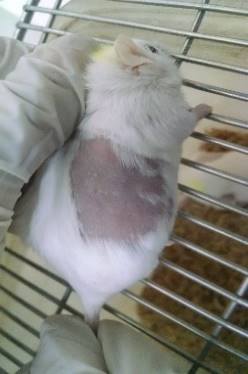

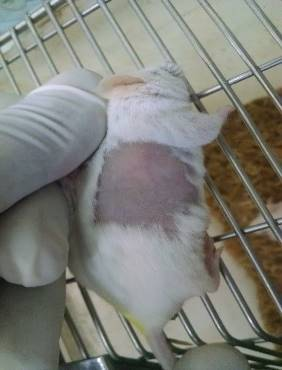

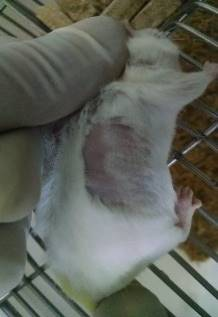

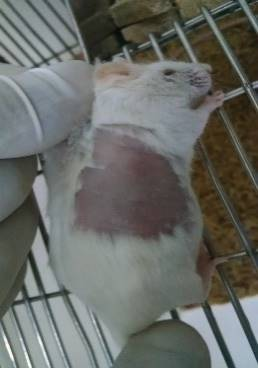

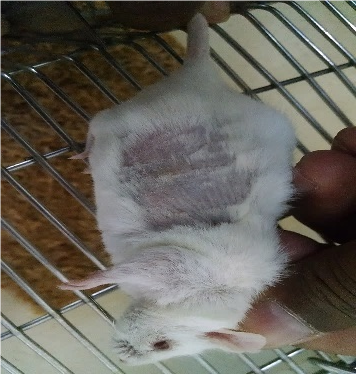

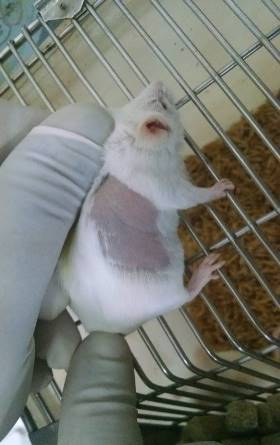

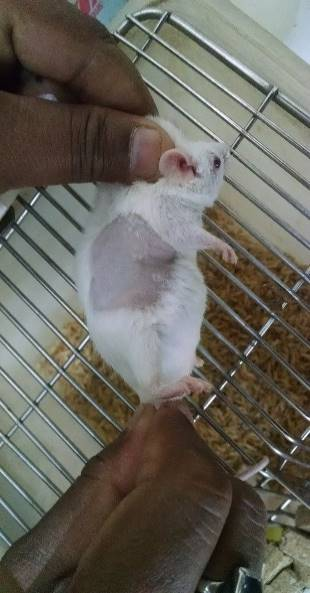

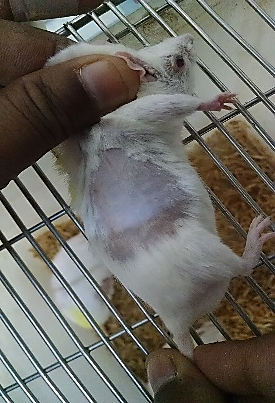


…………………………………..……..Day 14^th^……………………………………….

PII of LLA (2, 10 and 20%) was found to be in the category of irritation barely perceptible when studied in female and male mice. No significant change in body and organ weight was observed when compared with vehicle.

**Figure SM3:**

*Female rats Male rats*

Vehicle LLAS2% LLAS10% LLAS20% Vehicle LLAS2% LLAS10% LLAS20%


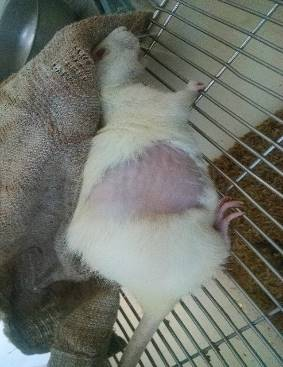

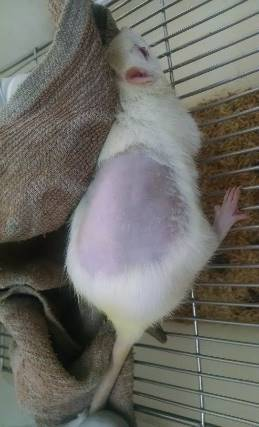

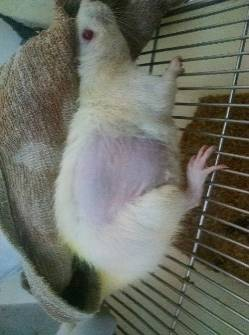

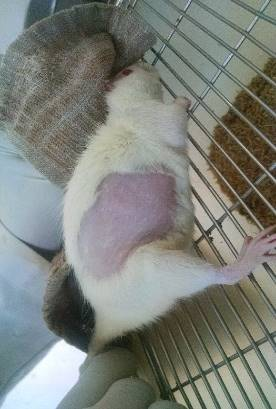

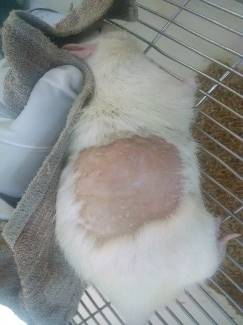

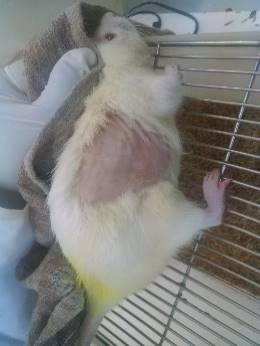

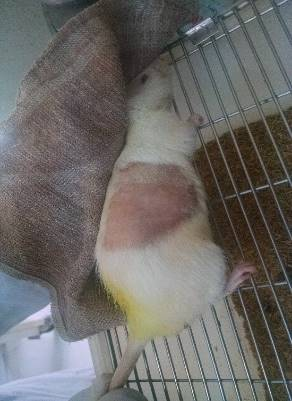

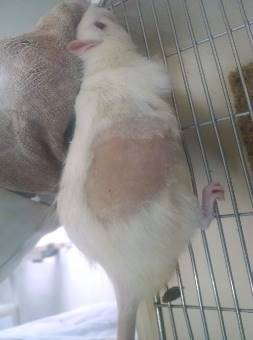


………………………………………….. Day 1^st^…………..………..………………….


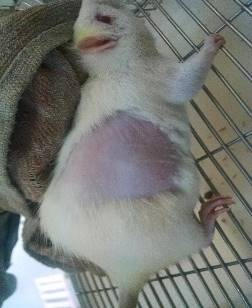

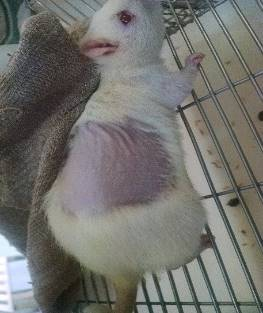

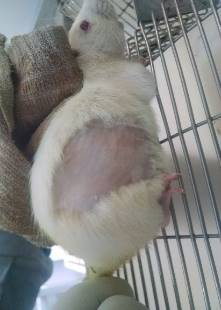

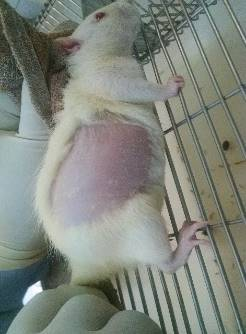

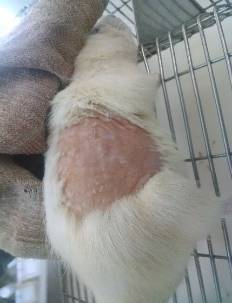

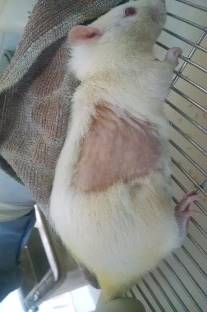

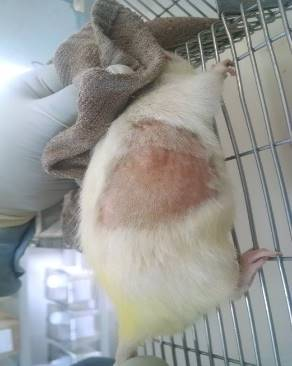

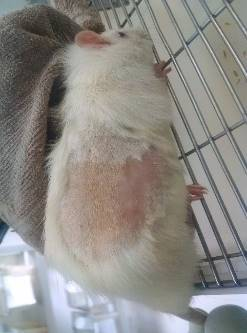


….……………………………………... Day 7^th^……………………………………….


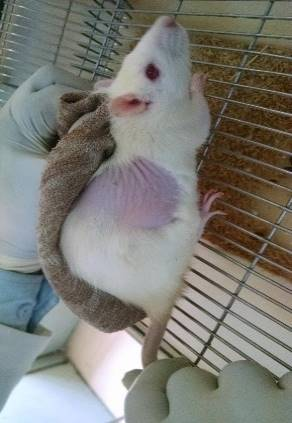

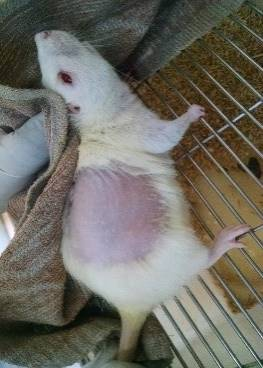

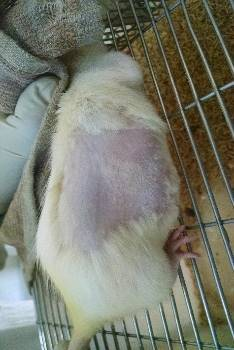

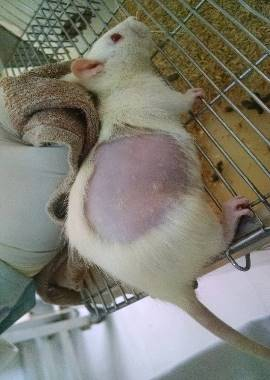

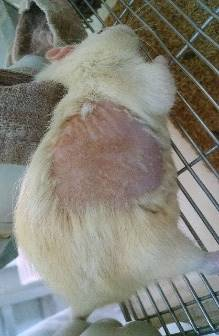

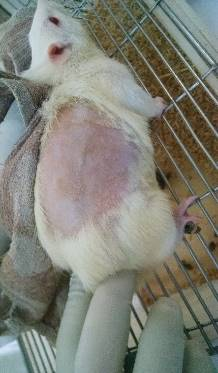

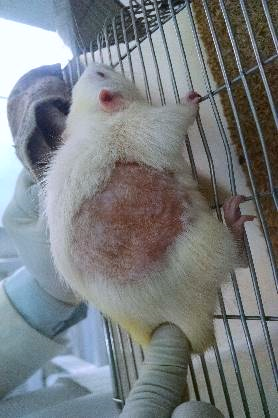

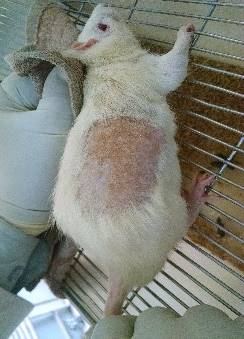


……………………………………..….. Day 14^th^……………………………………….

PII of LLA (2, 10 and 20%) was found to be in the category of irritation barely perceptible when studied in female and male rats. No significant change in body weight was observed when compared with vehicle treated animals.

**Figure SM4:**

*Female rats Male rats*

Vehicle LLA0.2% LLAS2.0% LLAS4.0% Vehicle LLA0.2% LLAS2.0% LLAS4.0%


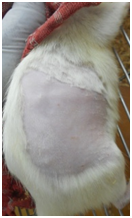

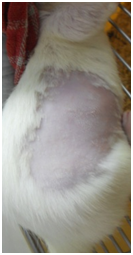

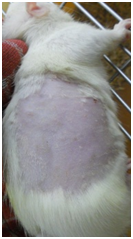

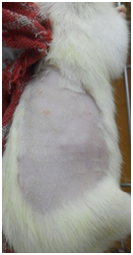

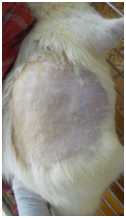

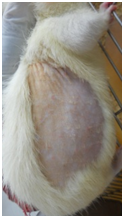

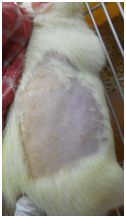

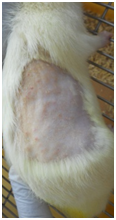


………………………………………….. Day 1^st^…………..………..………………….


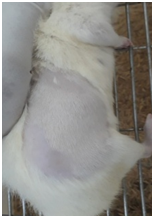

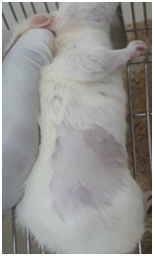

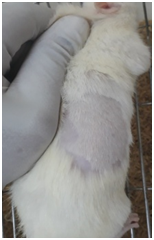

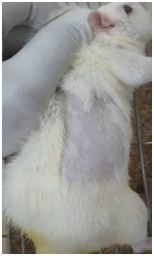

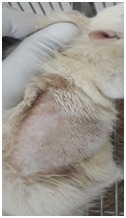

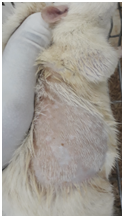

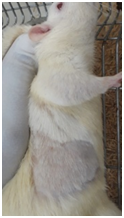

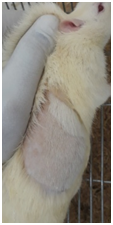


………………………………………….. Day 7^th^…………..………..………………….


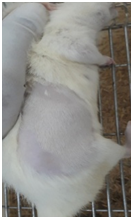

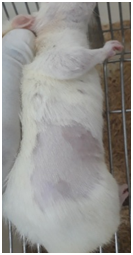

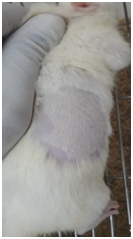

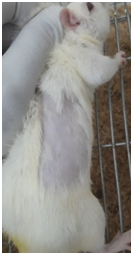

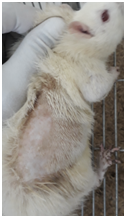

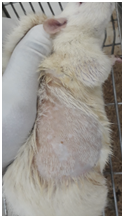

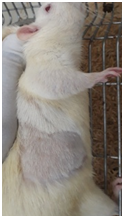

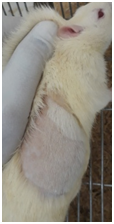


………………………………………….. Day 14^th^…………..………..………………….


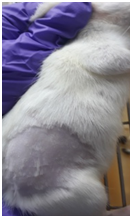

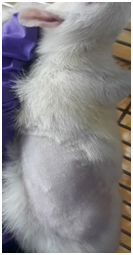

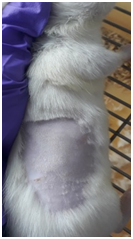

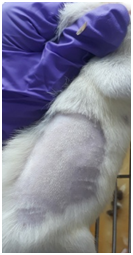

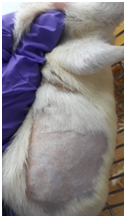

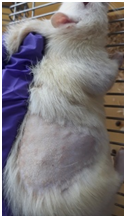

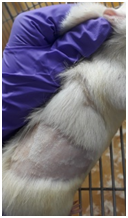

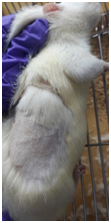


………………………………………….. Day 21^st^…………..………..………………….


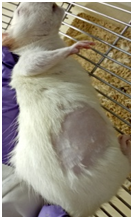

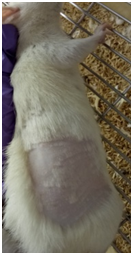

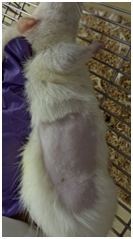

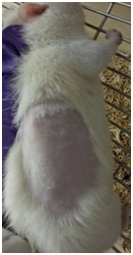

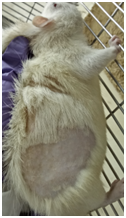

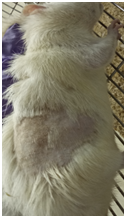

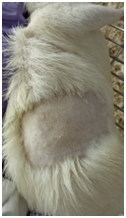

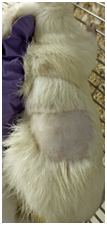


………………………………………….. Day 28^th^…………..………..………………….
