# Supplementary material for: A combination of linalool and linalyl acetate synergistically alleviates imiquimod-induced psoriasis-like skin inflammation in BALB/c mice
Source: Front Pharmacol. 2022 Aug 5;13:913174. doi: 10.3389/fphar.2022.913174 (PMC9388787; doi:10.3389/fphar.2022.913174)
Supplement: Supplementary file 7 [file Table4.DOCX]

Highlights

- The anti-psoriatic effect of lavender oil and its phytoconstituents was reported
- Linalool and linalyl acetate show distinctive affinity with various psoriasis targets
- Linalool and linalyl acetate synergistically alleviate psoriasis like inflammation
- Their combination does not show acute and repeated dose dermal toxicities
- This combination was able to achieve PASI 75 against IMQ induced psoriasis
